# Integrated Metagenomics and Metabolomics to Reveal the Effects of Policosanol on Modulating the Gut Microbiota and Lipid Metabolism in Hyperlipidemic C57BL/6 Mice

**DOI:** 10.3389/fendo.2021.722055

**Published:** 2021-10-11

**Authors:** Zhenya Zhai, Jianping Liu, Kai-Min Niu, Chong Lin, Yue Tu, Yichun Liu, Lichuang Cai, Huiping Liu, Kexian Ouyang

**Affiliations:** ^1^ Jiangxi Functional Feed Additive Engineering Laboratory, Institute of Biological Resource, Jiangxi Academy of Sciences, Nanchang, China; ^2^ Key Laboratory of Agro-Ecological Processes in Subtropical Region, Institute of Subtropical Agriculture, Chinese Academy of Sciences, National Engineering Laboratory for Pollution Control and Waste Utilization in Livestock and Poultry Production, Changsha, China; ^3^ Era Biotechnology (Shenzhen) Co., Ltd., Shenzhen, China

**Keywords:** policosanol, gut microbiota, metabolomics, C57BL/6 mouse, antihyperlipidemia

## Abstract

The aim of the study was to investigate the regulatory effects of policosanol on hyperlipidemia, gut microbiota and metabolic status in a C57BL/6 mouse model. A total of 35 C57BL/6 mice were assigned to 3 groups, chow (n=12), high fat diet (HFD, n=12) and HFD+policosanol (n=11), then treated for 18 weeks. Policosanol supplementation significantly reduced serum triglycerides and total cholesterol, as well as the weight of brown adipose tissue (BAT) (p<0.05), without affecting body weight in HFD-fed mice (p>0.05). Combined 16S rRNA gene sequencing and untargeted metabolomic analysis demonstrated that policosanol had regulatory effects on gut microbiota and serum metabolism in mice. In obese mice, policosanol increased the proportion of *Bacteroides*, decreased the proportion of *Firmicutes*, and increased the ratio of *Bacteroides* to *Firmicutes* (p<0.05). Policosanol promoted lipolysis and thermogenesis process, including tricarboxylic acid (TCA) cycle and pyruvate cycle, correlated with the increasing level of *Bacteroides*, *Parasutterella*, and decreasing level of *Lactobacillus* and *Candidatus_Saccharimonas.* Moreover, policosanol decreased fatty acid synthase (FAS) in the iWAT of obese mice. Policosanol also increased peroxisome proliferators-activated receptor-γ (PPARγ), uncoupling Protein-1 (UCP-1), peroxisome proliferator-activated receptor gamma coactivator-1α (PGC-1α) and PR domain containing 16 (PRDM16) in brown adipose tissue (BAT) obese mice (p<0.05). This study presents the new insight that policosanol may inhibit the synthesis of fatty acids, and promote lipolysis, thermogenesis related gene expression and regulate gut microbiota constituents, which provides potential for policosanol as an antihyperlipidemia functional food additive and provide new evidence for whole grain food to replace refined food.

## Introduction

With the gradual improvement of living standards of people in most countries, obesity induced by high-fat, high-sugar and refined diets is becoming a global problem (1). According to the World Health Organization, the number of obese people in the world nearly doubled between 1998 and 2008, with 9 million obese adolescents in the world (2). According to the data of the China Centers for Disease Control and Prevention, during 2013-2014, approximately 14% of Chinese people were also suffering from obesity (3). Hyperlipidemia, which mainly reflects the high cholesterol, high triglyceride content and lipid metabolism disorder in blood, is a major complication of obesity (4, 5). Long-term hyperlipidemia can induce nonalcoholic fatty liver and other diseases, which are harmful to human health.

The microbiome was found regulated by diets and has essential roles on impacting obesity by influencing the caloric absorption and energy expenditure (6). In germ-free mouse model, the bacteria transplantation dramatically increased the body weight and decreased the feed intake (7). However, a higher proportion of *Firmicutes* relative to *Bacteroidetes* was found in obese people and when obese people accepted a low-calorie diet and lose weight, this phenomenon was reversed (8, 9). Microbiome-metabolic axis plays important role in obesity (10). The metabolites of intestinal microbiota, such as bile acids, butyric acid, monosaccharide and vitamins, have been found to promote or ameliorate obesity (11, 12). For example, butyrate, a short-chain fatty acid produced by microbial degradation of carbohydrates, was found to upregulate downstream genes such as UCP1, PGC-1α and PRDM16 related to lipolysis and thermogenesis (13, 14). Secondary bile acids, metabolites of gut microbiota from primary bile acids, have been found to enhance the expression of downstream lipolysis and thermogenesis genes by activating bile acid receptors. There is growing consensus that the lipid metabolism and energy expenditure of the host can be regulated and improved by interfering the structure of intestinal microbiota.

Policosanol is a kind of long-chain fatty acid alcohols (LFAs) including octacosanol (C_28_H_57_OH, the most abundant component), tetracosanol (C_24_H_49_OH), triacontanol (C_30_H_61_OH) and tetratriacontanol (C_34_H_69_OH), that has been considered a functional food with hypolipidemic, antiobesity, antihypercholesterolemia, and antihypolipidemic activity and has been used as a potential adjuvant drug for type 2 diabetes mellitus in the past two decades (15). Policosanol exists widely in rice bran, beeswax and other natural products (15). However, compared with whole grain foods, policosanol in refined grains or related foods is almost completely lost (16). Policosanol has been proven to be absorbed easily, and metabolized by animals and to exert activity on blood lipids. In a mouse model, octacosanol can be absorbed after oral administration, the plasma peak was reached in 30-60 min and existed in the body for more than 3 hours. is mainly concentrated in the liver, adipose tissue and digestive tract (17). Furthermore, octacosanol, the main component of policosanol, has been reported to reduce blood cholesterol levels, insulin resistance, and low-density lipoprotein cholesterol (LDL-C) levels after oral administration in humans (18). Similarly, in children with hypercholesterolemia, oral administration of policosanol can significantly reduce the levels of TC, LDL and apolipoprotein B (19). Furthermore, in a rat model and in Hep G2 cell models, octacosanol has been found to inhibit cholesterol synthesis (20). These studies provide clues that policosanol can regulate blood lipid and cholesterol levels and is targeted mainly to the liver and fat tissues, however, whether policosanol has a regulating effect on gut microbiota and serum metabolism is still no clear.

To clarify this situation, we attempted to investigate the regulatory effects of policosanol on hyperlipidemia, gut microbiota and serum metabolic status in mice by integrated microbiome-metabolomic methods, coupled with serum biochemical and gene expression analyses in the present study.

## Materials and Methods

### Ethics Statement

All the experimental design, procedures and experimental operations in the present study were approved and in accordance with the guidelines of the Committee of the Institute of Subtropical Agriculture at the Chinese Academy of Science (No. ISA-2020-18).

### Preparation of Policosanol

Policosanol was prepared by a distillation method. Briefly, we constructed a vacuum high-temperature distillation column. The saponified crude alcohol used as raw material was added into the material tank, high-temperature distillation was carried out in the distillation column, and policosanol with different purities was collected in different collectors. The Analyzing and Testing Center of Guangzhou Institute of Chemistry, Chinese Academy of Sciences, was entrusted to test the components of policosanol (report number: YS160503-03). The policosanol used in this study was composed of docosanol (C_22_H_45_OH, 2.48%), tetradecanol (C_24_H_49_OH, 4.19%), hexacosanol (C_26_H_53_OH, 4.33%), octacosanol (C_28_H_57_OH, 64.16%) and triacontanol (C_30_H_61_OH, 15.51%). Because policosanol is a waxy solid, it is necessary to use a vehicle to improve its mixing uniformity in the diet. The vehicle includes mainly cyclodextrin, isomaltooligosaccharide and Arabic gum. The ratio of vehicle and policosanol was 1:9 (weight: weight).

### Animals

A total of 35 male C57BL/6 specific pathogen-free mice (3-4 weeks of age) were purchased from STJ Laboratory Animal Co., Ltd. (Hunan, China). All mice were housed in cages and raised under the same controlled conditions (temperature 25 ± 2°C, light/dark 12 h:12 h, humidity of 60 ± 10%). After 1 week of adaptation, the mice were randomly assigned to 4 groups with equally adjusted initial body weights as follows: the chow group was fed a chow diet containing vehicle (17.81 ± 0.16 kJ/g, n=12), the HFD group was fed an high-fat diet containing vehicle (24.02 ± 0.09 kJ/g, n=12), and (4) the HFD+policosanol group was fed HFD containing 0.5% policosanol (23.97 ± 0.06 kJ/g, n=11). All the diets were customized by Jiangsu Xietong Pharmaceutical Bio-Engineering Co., Ltd. The normal and HFD formulas are shown in **Supporting Information Tables S1**, **S2**. All the mice were raised with free access to feed and water and body weight and food intake of the mice were measured once a week for 18 weeks.

### Sample Preparation

At the end of this experiment, all the mice were fasted for 6 h, and anesthesia was induced by intraperitoneal injection of 2% pentobarbital sodium intraperitoneally injection (45 mg/kg body weight). Then, blood samples were collected after enucleation of the eyeball. Then, the inguinal white adipose tissue (iWAT), epididymal white adipose tissue (eWAT) and brown adipose tissue were separately collected and weighed, as described in our previous study (12, 21). iWAT was completely separated from the subcutaneous of the abdomen. Further open the abdominal cavity and completely separate eWAT from around the epididymis. Finally, the skin of the scapula was cut to completely separate the brown fat. The iWAT samples were placed into 4% paraformaldehyde and embedded in paraffin and then subjected to hematoxylin and eosin (H&E) staining. Adipocyte size was measured using VistarImage (Olympus, Japan) following the instructions. The blood, adipose tissue and cecal content samples for metabolomics analysis, qPCR or 16s rRNA were collected with centrifuges (germ-, RNase- and DNase- free) tube, frozen in liquid nitrogen and stored at - 80 °C.

### Lipid Parameters in Serum

The triglyceride (TG), total cholesterol (TC), high -density lipoprotein cholesterol (HDL-C) and LDL-C in serum were determined according to the instructions of the commercial kits (Nanjing Jiancheng, Bioengineering Institute, Nanjing, China).

### 16S rRNA Gene Sequencing

Detailed sequencing methods were described in our previous research (22) and performed in a commercial company (Novogene, Beijing, China). Briefly, total DNA was extracted from the cecum contents by using Qiangen QIAamp DNA Stool Mini Kit according to the protocol. Then the V3-V4 region of the bacterial 16S ribosomal RNA gene amplified by PCR, the following primers (5’-3’) 180 were used: 341F CCTAYGGGRBGCASCAG and 806R 181 GGACTACNNGGGTATCTAAT. After the PCR, and the amplicons were electrophoresed and extracted. The gene library was constructed by using the Ion Plus Fragment Library Kit 48 rxns 188 (Thermo Scientific, Waltham, MA, USA). Then the sequencing of genes was performed on the Ion S5™XL platform to obtain the raw data. The raw data were treated and measured to clean data. The clean data were clustered with 97% identity to identify the operational taxonomy units (OTUs).

Linear discriminant analysis (LDA) effect size (LEfSe) was used to elucidate the differences in bacterial taxa, which were described in our previously study (22). An LDA score ≥3 was considered to be an important contributor to the model. Spearman analysis was used to measure the correlation between gut microbiota and adipose tissue weight or serum metabolites using R software.

### Untargeted Metabolomics in Serum

Untargeted metabolomics was conducted using a commercial service from Biotree (Shanghai, China). Briefly, a 50 μL serum sample was mixed with 200 μL extracting solution (50% methanol: 50% acetonitrile) and internal standards [L-leucine-5,5,5-d3 (CAS:87828-86-2), trimethylamine-d9-N-oxidein (CAS: 1161070-49-0)] in a 1.5 mL centrifuge tube. The mixture was sonicated in an ice-water bath for 10 min and placed at -40°C for 1 h. Then, the samples were centrifuged at 4°C and 12000 rpm for 5 min, and the supernatant was subjected to liquid chromatography-mass spectrometry (LC-MS).

The LC was performed using a Vanquish ultra-performance liquid chromatography instrument (Thermo Fisher Scientific, MA, US) with a Waters ACQUITY UPLC BEH Amide (2.1 mm × 100 mm, 1.7 μm) chromatographic column to separate the metabolites. A phase (ultrapure water containing 25 mmol/L ammonium acetate and 25 mmoL/L ammonia) and B phase (acetonitrile) were used for elution. The elution gradient is shown in **Supporting Information (Table S2)**. The column temperature was 30°C, and the injection volume was 3 μL.

A QE HFX mass spectrometer was used to obtain MS/MS spectra in information-dependent acquisition (IDA) mode under the control of the acquisition software (Xcalibur, Thermo Fisher Scientific). In this mode, the acquisition software continuously evaluates the full scan MS spectrum. The ESI source conditions were set as follows: sheath gas flow rate of 50 Arb, Aux gas flow rate of 10 Arb, capillary temperature of 320°C, full MS resolution of 60000, MS/MS resolution of 7500, collision energy of 10/30/60 in NCE mode, and spray voltage of 3.5 kV (positive) or -3.2 kV (negative).

The raw data were converted to an mzXML format, and R software was used for peak detection, extraction alignment and integration. The metabolites were annotated using an in-house database. The peaks were normalized using internal standard.

### Bioinformatic Analysis

To analyze the differences in gut microbiota and serum metabolites in different groups, principal component analysis (PCA), and orthogonal partial least squares discriminant analysis (OPLS-DA) were performed and the over fitting of the model is verified by cross-validation (permutation test) using SIMCA-P (16.0.2, Sartorius Stedim Data Analytics AB, Umea, Sweden) (23). Then, univariate statistical analysis was used to screen metabolic markers with significant differences in accordance with our previous study (24, 25). Briefly, based on OPLS-DA, the variable importance in the projection (VIP)>1, the relative abundance fold change of the metabolites of <0.5 or >2, and a p-value <0.05 were defined as differences. Furthermore, the significantly changed metabolite pathways were analyzed using the KEGG database (Kyoto Encyclopedia of Genes and Genomes).

### RNA Extraction and Quantitative Real-Time Polymerase Chain Reaction

The iWAT and BAT were homogenized and total mRNA was extracted using column RNA extraction kits (Magen, Guangzhou, China, R4121). The total RNA concentration was determined using a NanoDrop 2000C spectrophotometer (Thermo Fisher Scientific, Waltham, MA, USA), and the mRNA was reverse transcribed to cDNA using cDNA synthesis kits (CWBIO, Jiangsu, China, CW2582M). Information on the primers is shown in **Supporting Information (Table S6)** and synthesized (Sangon Biotech, Shanghai, China). qPCR was performed in an Applied Biosystems by Life Technologies QuantStudio 7 (Thermo Fisher Scientific). The relative abundance of the genes was normalized to GAPDH using the 2-ΔΔCT method and the relative expression level were shown as fold changes relative to chow group.

### Statistical Analysis

Data are expressed as the mean ± SEM. One-way ANOVA and least significance difference (LSD) method as a suitable *post-hoc* test was used to determine the differences among the groups by using SPSS 20.0 (IBM, SPSS, USA) and a p-value of <0.05 was considered statistically significant. GraphPad Prism 7 (GraphPad Software Inc., San Diego, CA, USA) was used to generate statistical plots.

## Results

### Effects of Policosanol on Hyperlipidemia in HFD-Fed Mice

**Figure 1:**
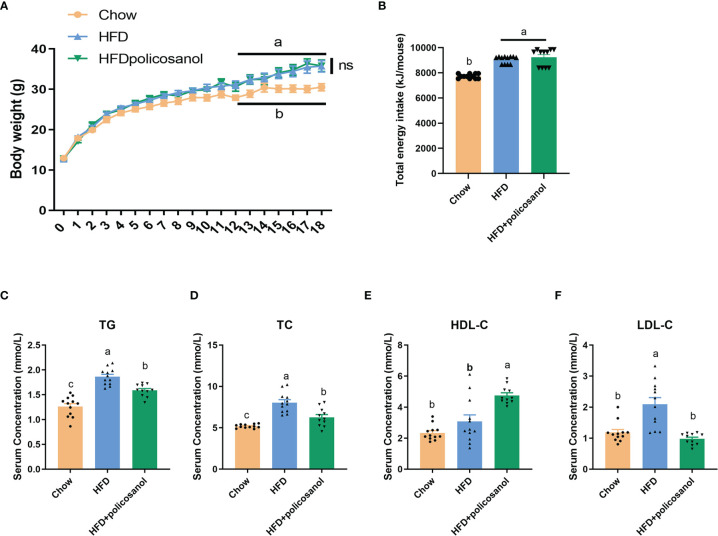
Policosanol did not affect body weight, but showed the lipid lowering effect in the serum of mice. **(A)** Policosanol had no effect on the body weight of obese mice for 18 weeks. **(B)** The total energy intake (per mice) of chow diet or HFD fed mice after 18 weeks. **(C–F)** Policosanol reduced serum TG, TC, LDL-C, HDL-C in HFD induce obese mice for 18 weeks. Chow: The mice fed with chow diet (n=12), HFD: the mice fed with high fat diet (n=12), HFD+policosanol: The mice fed with high fat diet containing 0.5% policosanol (n=11). TG, Total glyceride; TC, total cholesterol; LDL-C, low density lipoprotein cholesterol; HDLC, high density lipoprotein cholesterol. Data were expressed as mean ± SEM. Differences of data in mice subjects were assessed by one-way ANOVA. For all pictures, columns indicated with different letters (a- c) have significant difference, p < 0.05. ns, not significant.

C57BL/6 male mice aged 3-4 weeks were fed 0.5% policosanol in a chow diet or HFD for 18 weeks. The data showed that the body weight of HFD-fed was mice significantly higher than the body weight of chow-fed mice (p<0.05). However, policosanol treatment did not significantly affect changes in body weight, or total energy intake in HFD-fed mice (p>0.05) (**Figures 1A, B**). In serum, the TG and TC contents were both significantly reduced in the HFD-fed mice (p<0.05, **Figures 1C, D**), while decreased LDL-C content and increased HDL-C content were observed in HFD-fed mice (p<0.05, **Figures 1E, F**) after policosanol treatment.

### Effects of Policosanol on Body Fat in HFD-Fed Mice

**Figure 2:**
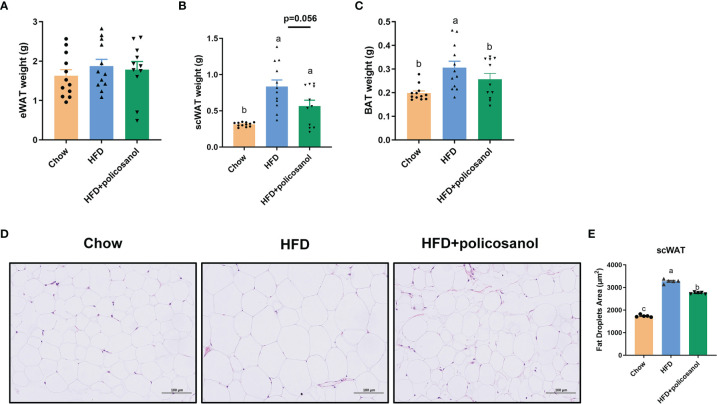
Policosanol reduced lipid accumulation in the iWAT and BAT. **(A–C)** The tissue weight of eWAT, iWAT and BAT. Chow: The mice fed with chow diet (n = 12), HFD: the mice fed with high fat diet (n = 12), HFD + policosanol: The mice fed with high fat diet containing 0.5% policosanol (n = 11). **(D)** Representative H&E staining picture of adipocyte size of iWAT. **(E)** Statistical plot of fat droplet area in scWAT, n = 5 mice/group. Differences of data in mice subjects were assessed by one-way ANOVA. For all pictures, columns indicated with different letters (a-c) have significant difference, p < 0.05. ns, not significant.

The accumulation of adipose tissue in mice was evaluated. Policosanol treatment showed no effect on eWAT weight (p>0.05, **Figure 2A**), tended to reduce the weight of iWAT (p=0.056, **Figure 2B**), and significantly decreased the BAT weight (p<0.05) in the HFD-fed mice (**Figure 2C**). H&E staining-based histological results for both groups showed that policosanol reduced the iWAT adipocyte size (p<0.05, **Figures 2D, E**).

### Effect of Policosanol on Gut Bacterial Communities in Mice

**Figure 3:**
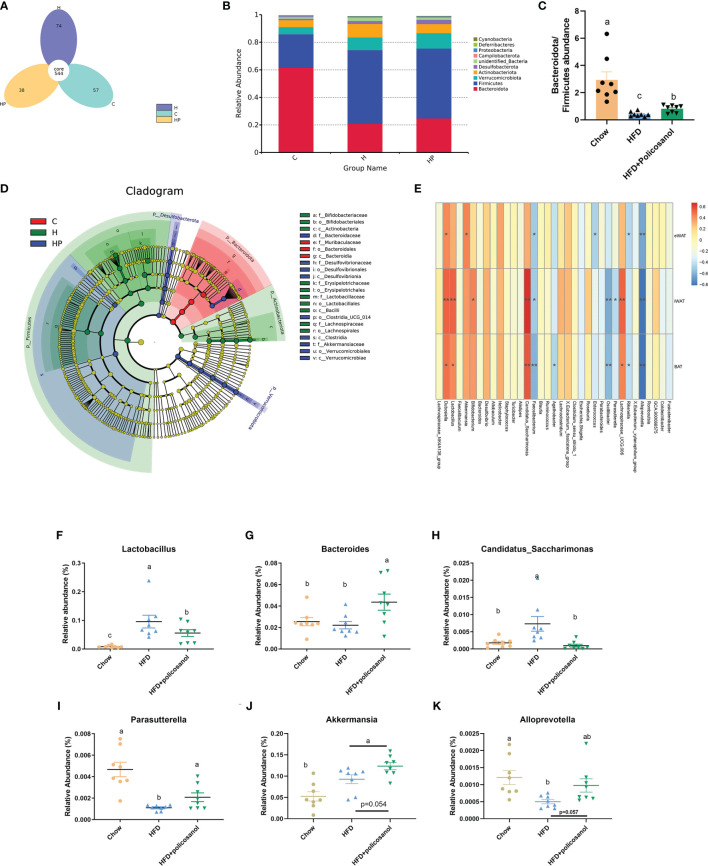
Policosanol reshaped the gut microbiota in mice. **(A)** The Venn plot of OTUs. **(B)** Relative abundance of gut microbiota at phylum level. **(C)** The of *Bacteroides*/*Firmicutes* ratio in the cecal contents of mice. **(D)** LEfse analysis between HFD and HFD+policosanol groups. **(E)** The correlation among microbiota and adipose tissue weight. ** : | R^2^| > 0.5, *0.4 < | R^2^| < 0.5 **(F–K)** The key microbiota with significant difference at genus level. **(C)** mice fed with chow diet. **(H)** mice fed with HFD. HP: mice fed with HFD+0.5% policosanol diet, n=8/mice per group. Differences of data in mice subjects were assessed by one-way ANOVA. For all pictures, columns indicated with different letters have significant difference (a-c), p < 0.05. ns, not significant.

The16S rRNA gene sequencing data are shown in **Figure 3**. The rarefaction and observed OTU results showed that the selective sequences in clean data were sufficient to determine most of the bacterial species (**Supporting Information Figures S1 A, B**). The petal diagram showed that there were 544 core OUTs found in all the groups, while 57, 74 and 38 OTUs were detected in chow, HFD and HFD+policosanol groups (**Figures 3A** and **S1C**). Policosanol increased *Bacteroides*, decreased the *Firmicutes* relative abundance, and increased the B/F ratio in obese mice (p<0.05, **Figures 3B, C**). To better understand the effect of policosanol on microbiota constituents, LEfse was conducted (**Figure 3D**). The data showed that *Bacteroidales* and *Muribaculaceae* belonging to *Bacteroidota* phylum were the feature taxa in the chow fed mice. In HFD group, *Lactobacillaceae*, *Actinobacteria* and *Erysipelotrichales* were the feature taxa. In HFD+policosanol group, *Akkermansiaceae* belonging to *Verucomicrobiales*, *Bacteroidaceae* and *Desulfovibrionia* were the feature taxa. Based on this, a spearman correlation analysis was conducted between microbiota composition and adipose tissue weight. The data showed that *Lactobacillus*, *Dubosiella*, *Candidatus:Sarccharinonasm* and *Lachnospiaceae_UGG.006* showed positive correlation with iWAT and BAT weight (R^2^>0.4), while *Alloprevotella*, *Parasutterella*, *Oscillibacter* and *Faecalibacterium* showed negative correlation (R^2^<-0.4). Furthermore, compared to HFD group, policosanol was found had lowering-effect on the relative abundance of *Lactobacillus* and *Candidatus:Sarccharinonasm* compared to HFD group (**Figures 3F, G**, p<0.05), while up-regulating effect on the relative abundance of *Bacteroides* and *Parasutterella* (p<0.05, **Figures 3I, J**). It is also found that, compared to HFD group, policosanol treatment tend to increase the relative abundance of *Allpprevotella* and *Akkmermansia* (0.05<P<0.1, **Figures 3J, K**).

### Effects of Policosanol on Serum Metabolites in HFD-Fed Mice Uncovered by Untargeted Metabolomics

**Figure 4:**
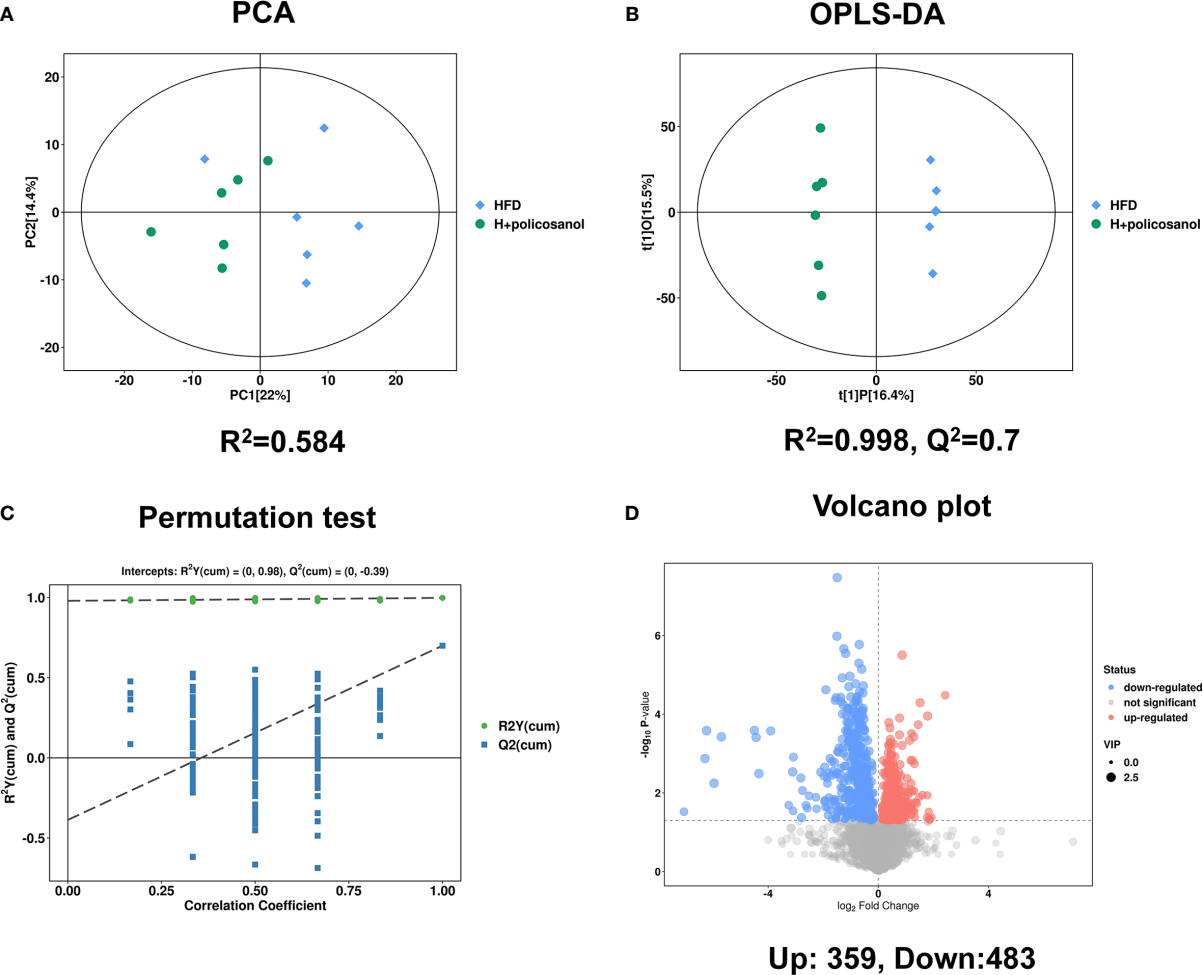
Policosanol affects the metabolites in mice. **(A)** The principal component analysis plot based on the metabolites matrix in the serum. **(B)** The orthogonal partial least squares discrimination analysis (OPLS-DA) plot based on the metabolites matrix in the serum, **(C)** permutation test plot based on OPLS-DA methods. **(D)** The volcano plot based on the changed metabolites the HFD group compared to the HFD+policosanol group.

Untargeted metabolomics with both positive and negative models was subsequently conducted to investigate the effects of policosanol on influencing serum metabolite changes related to lipid and energy metabolism. The PCA and OPLS-DA results showed discriminately distinguished serum metabolites in HFD-fed mice (**Figures 4A, B**). The permutation test showed that all OPLS-DA models were reliable without overfitting (**Figure 4C**). The volcano plot (**Figure 4D**) presented significantly changed serum metabolites after treatment with policosanol. In HFD-fed mice, we found that 483 metabolites were decreased and 359 metabolites were increased after policosanol treatment.

### Effects of Policosanol on Changing the Metabolic Pathways in HFD-Fed Mice

**Figure 5:**
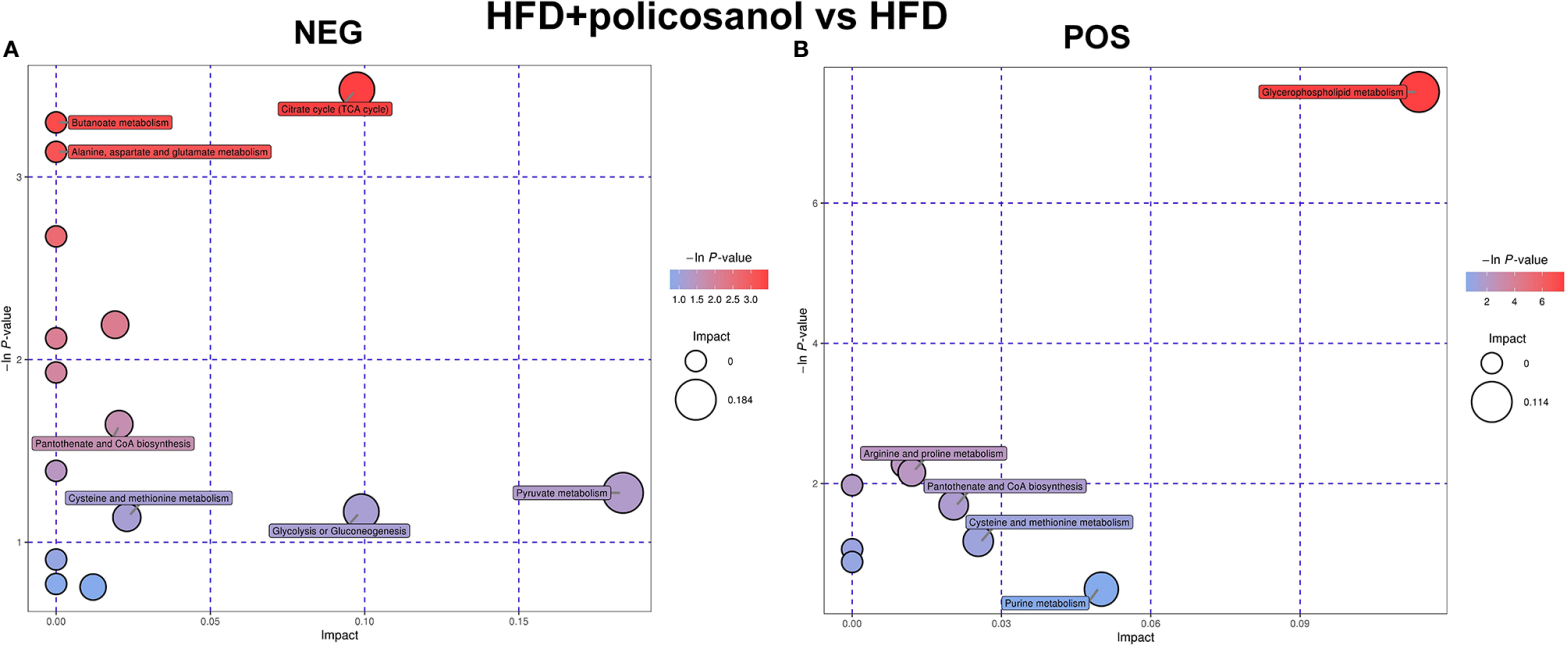
Policosanol regulates the metabolic pathways in obese mice. **(A)** Enrichment analysis of KEGG pathways in NEG mode. **(B)** Enrichment analysis of KEGG pathways in POS mode. NEG, negative scanning mode; POS, positive scanning mode.

Based on the PCA, the relative abundance of metabolites between HFD and HFD + policosanol mice were shown to be remarkably different. Thus, relevant metabolic pathways were further analyzed (**Figure 5**). In HFD-fed mice, policosanol was found to regulate mainly the TCA cycle, butanoate metabolism, alanine, aspartate and glutamate metabolism, pyruvate metabolism, pantothenate and CoA biosynthesis and glycerophospholipid metabolism pathways under the negative scan model, while the regulation related to glycerophospholipid metabolism, arginine and proline metabolism, and cysteine and methionine metabolism-related pathways was determined under the positive the scan model. Small molecule metabolites in mouse cecal contents were also detected. The data showed that policosanol treatment mainly affect the linoleic acid metabolism, biosynthesis of unsaturated fatty acids, propanoate metabolism and amino acid metabolism (**Supplementary Figure S2**).

### Effects of Policosanol on the Regulation of Representative Metabolites in Chow- and HFD-Fed Mice

**Figure 6:**
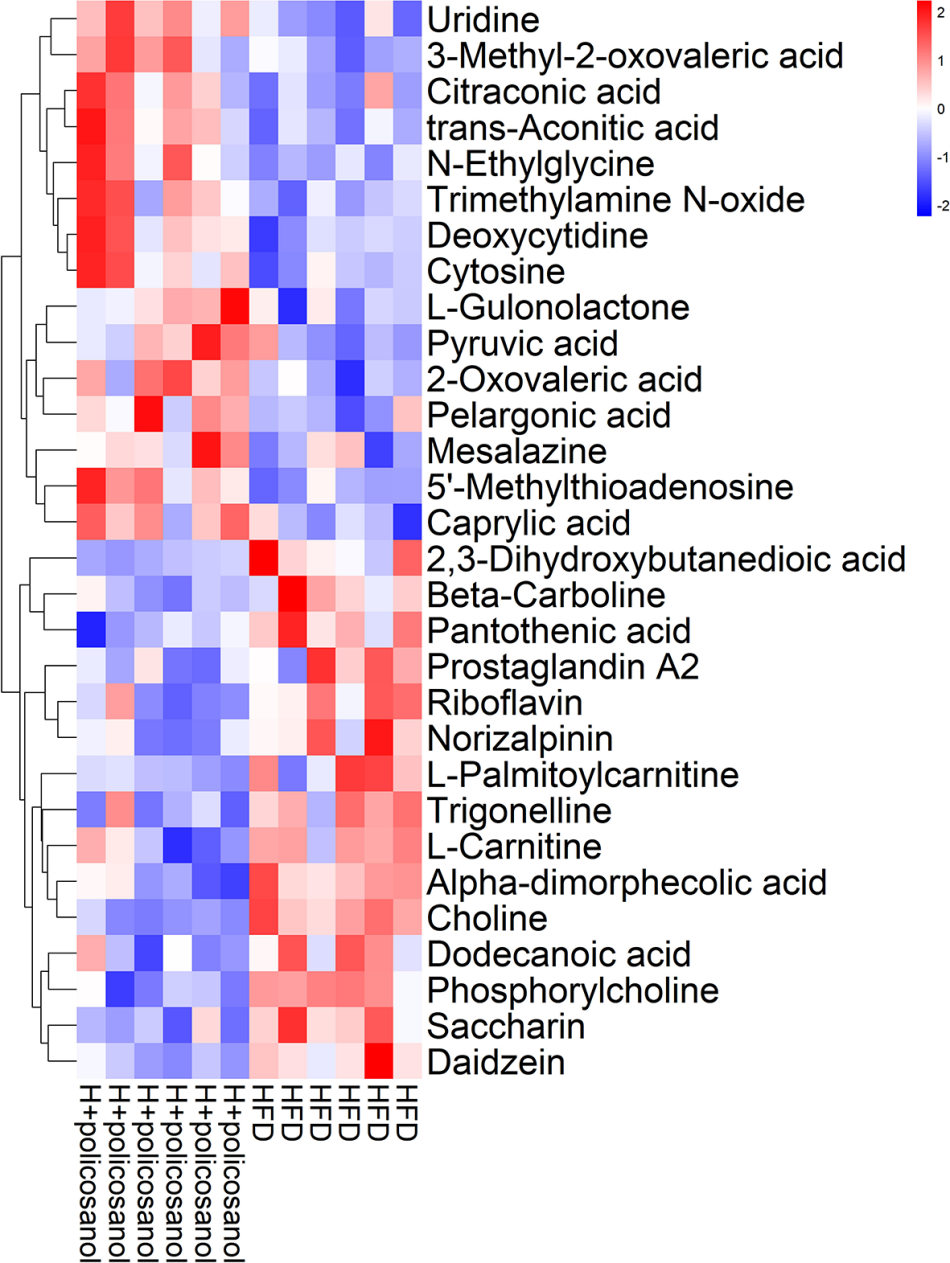
Hierarchical clustering analysis of top the 30 metabolites in mice. Heat map of top the 30 metabolites in HFD and HFD +policosanol groups. The red and blue indicate upregulation and downregulation, respectively.

Further, we combined OPLS-DA, t-test and serum content to screen key small molecule metabolites that may be regulated by policosanol. A total of 30 metabolites were primarily identified (**Figure 6**) and all the significantly changed metabolites found in this study were shown in **Supporting Information Tables S5**.

In HFD-fed mice, metabolites involved in nucleotides, amino acids, fatty acids, and their intermediates that are associated with mitochondrial oxidation, such as 3−methyl−2−oxovaleric acid, citraconic acid, trans−aconitic acid, and pyruvic acid, were significantly enriched with policosanol treatment compared to the control. Conversely, glycerophospholipid metabolism-related metabolites such as 2,3−dihydroxybutanedioic acid, pantothenic acid, L−carnitine, choline, phosphorylcholine, and trimethylamine oxide (TMAO), were significantly downregulated. The data showed that, policosanol significantly decreased the content of lipids and lipid-like molecules (e.g., 12-HEPE and 12-HETE, the derivates of eicosapentaenoic acid) and increased the content of linoleic acid and ricinoleic acid (p<0.05, **Supporting Information Figure S3** and **Supplementary Table S6**) in the cecal content of mice.

### Correlation of Key Microbiota Communities and Differential Serum Metabolites

**Figure 7:**
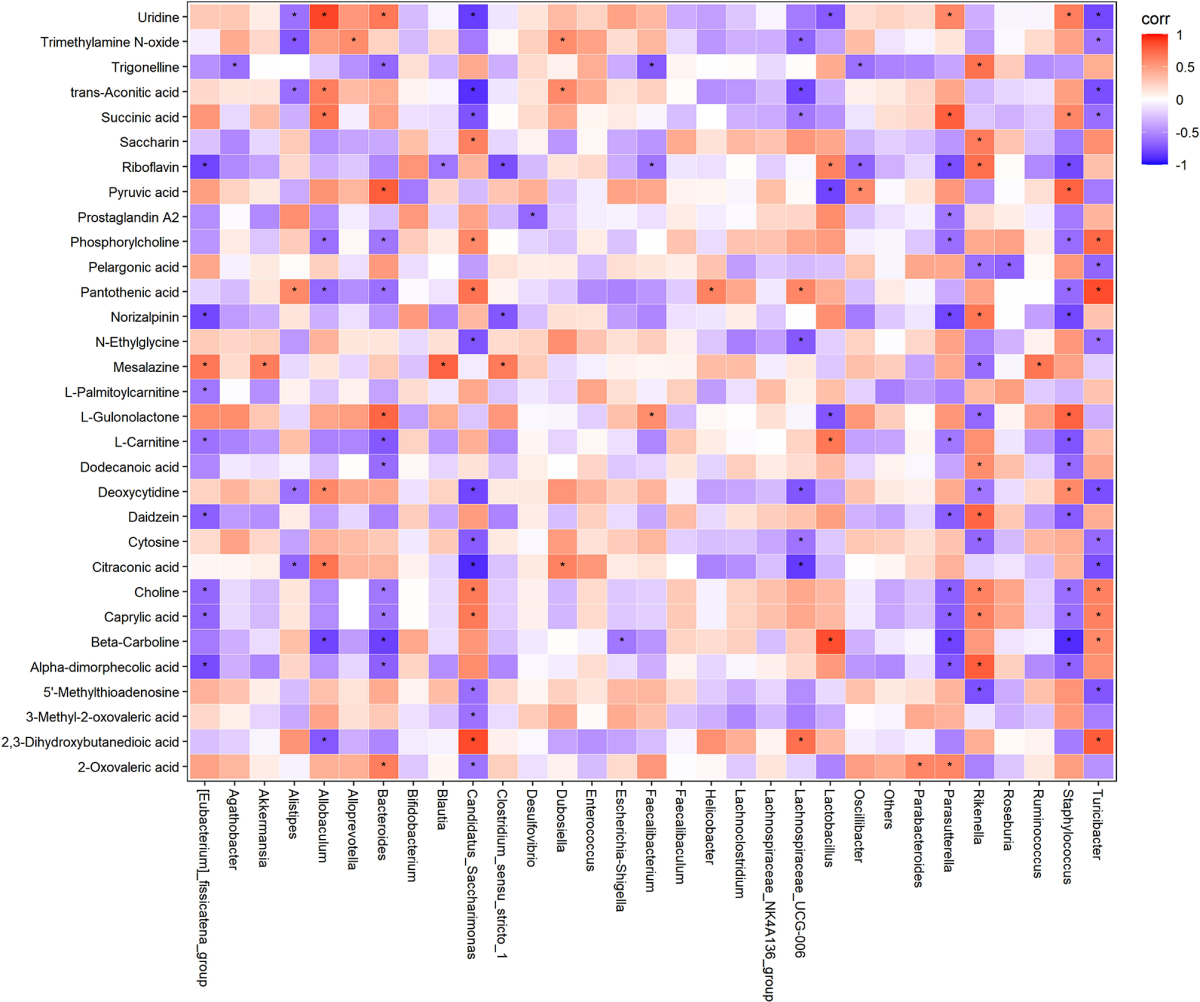
Correlation analysis of gut microbiota and serum metabolites in HFD and HFD+policosanol mice. Red and blue indicate positive and negative correlations, respectively. *p < 0.05.

To better reveal how altered microbiota regulate host metabolic processes in mice, correlation analysis of microbiome-metabolomic studies were conducted in obese mice (**Figure 7**). In HFD induced obese mice, *Allobactulum* is the feature taxa in HFD + policosanol, was positively correlated with the metabolites involved in energy metabolism, such as succinic acid, trans-aconitic acid and citraconic acid. *Bacteroides* were positively correlated with pyruvic acid and uridine (R^2^>0.4, p<0.05) but negatively correlated with trigonelline, phoshorycholine, pantothenic acid, L-cartnitine (R^2^<-0.4, p<0.05). In contrast, *Candidatus_Sacchanimonas* and *Turicibacter*, the feature communities in the HFD group, were negatively correlated with uridine, trans-aconitic acid, succinic acid, citraconic acid (R^2^<-0.4, p<0.05), while positively correlated with choline, pantothenic acid and 2,3-dihydroxybutanedioic acid (R^2^>0.4, p<0.05). Inductive information on the sources of all small molecule metabolites is presented in **Supporting Information Table S7**.

### Effects of Policosanol on the Regulation of Lipid Metabolism-Related mRNA Expression in Adipose Tissue

**Figure 8:**
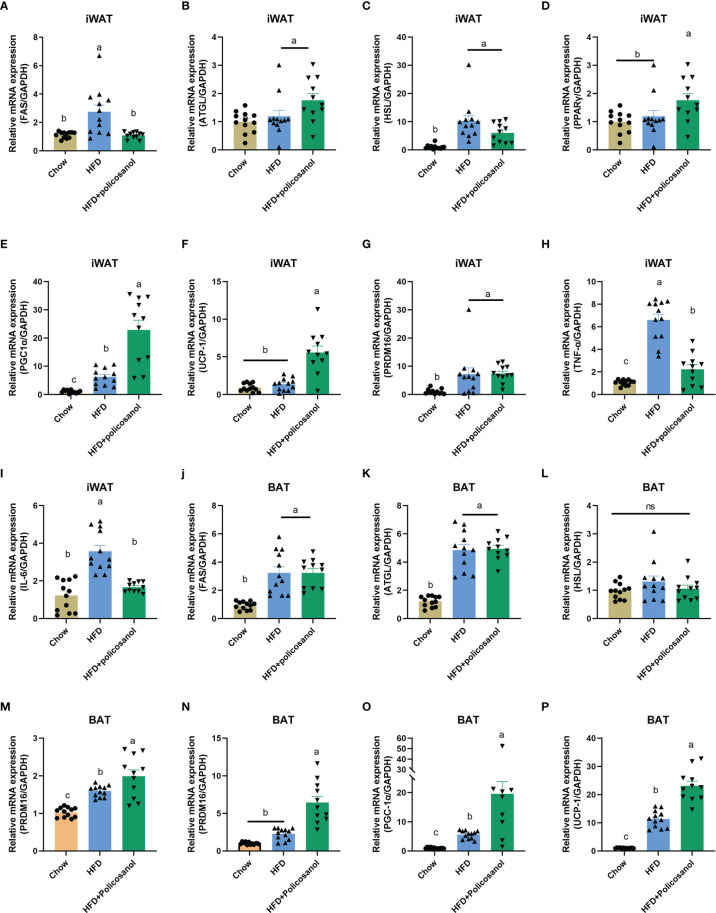
Policosanol activates AMPK and downstream gene expression related to fatty acid synthesis, inflammation, lipolysis and thermogenesis in adipose tissue. **(A–I)** Relative mRNA expression in iWAT. **(J–P)** Relative mRNA expression in the BAT. Chow: The mice fed with chow diet (n=12), HFD: the mice fed with high fat diet (n=12), HFD+policosanol: The mice fed with high fat diet containing 0.5% policosanol (n=11). Differences of data in mice subjects were assessed by one-way ANOVA. For all pictures, columns indicated with different letters have significant difference, p < 0.05. ns, not significant.

In iWAT, policosanol treatment decreased the gene expression of FAS, TNF-α and IL-6, while increased PPARγ, PGC-1α and UCP-1 in HFD-fed mice (p<0.05, **Figures 8A**–**I**). Similarly, in BAT, policosanol showed no significant effect on FAS, ATGL, HSL, (p>0.05), but upregulation of PPARγ, UCP1, PGC-1 and PRDM16 in obese mice after policosanol treatment (p<0.05, **Figures 8J**–**P**).

## Discussion

Obesity is a chronic metabolic disease that has been found to be closely related to cardiovascular disease, diabetes and gut disease since the early 20th century. To date, the causes of obesity are still unclear, but controlling energy intake, increasing energy consumption and promoting fat decomposition and oxidation have been deemed to alleviate obesity (26). In general, pharmacotherapy intervention, bariatric surgery treatment and lifestyle intervention are mainly used to treat obesity (27). However, these interventions can cause side effects such as vitamin deficiency, hyperthyroidism, headache, vomiting and hypoglycemia (28, 29). Among these interventions, lifestyle interventions, including adjustment of dietary structure and exercise, are considered safe ways to improve obesity. A large amount of evidence has shown that policosanol improves hypertriglyceridemia and hypercholesterolemia in humans and animals and has the potential as a functional food to improve obesity. For example, a double-blind experiment on volunteers with prehypertension showed that orally taking 10 mg policosanol tablets (containing 60-70% octacosanol) each day can reduce TC and TG by 20% and 14%, respectively, and the activity of cholesteryl ester transfer protein in the serum (30). Policosanol (mainly containing 28% octacosanol, 21% triacontanol, and 36% tetratriacontanol) also significantly decreased the weight of iWAT and BAT (31). The present study was conducted to investigate the effects of policosanol on metabolism *in vivo* and to explore the potential mechanisms for improving obesity. Our results showed that dietary supplementation with 0.5% policosanol (containing approximately 64% octacosanol) did not change the body weight or total energy intake of mice. This is consistent with a previous study that showed that policosanol orally administered for 6-12 months does not affect the body weight of Sprague-Dawley rats (32, 33). Similarly, octacosanol, the main component of policosanol, has no effect on mouse body weight after treatment for 12 weeks (34). However, the present study found that policosanol can effectively reduce the content of TG, TC, LDL-C and increase HDL-C content in HFD-fed mice, suggesting its potential to ameliorate hyperlipidemia.

The diversity, richness and structure of gut microbiota play an important role in the regulation of host obesity, diabetes and metabolic diseases. The individuals who were marked as overall adiposity, were found with low bacterial richness compared to the non-obese individuals (35). In the present study, we analyzed the effects of policosanol on gut microbiota in obese mice. Policosanol reduced the weight of iWAT, BAT but not eWAT. Then, the effect of policosanol was further confirmed by histological analyses showing reduced adipocyte size in iWAT. As expected, the gut microbiota of mice fed with diet (containing 0.5% policosanol) for 18 weeks changed significantly and showed change manners in obese mice. Policosanol increased *Bacteroides*/*Firmicutes* ratio only in obese mice. In addition to the proportional changes of *Bacteroidea* and *Firmicutes*, the significant change of *Parasutterella* and *Canaidatus_Saccharimonas* after policosanol intervention was highly correlated with the weight change of iWAT and BAT. *Akkermansia* and *Alloprevotella* tended to recover to a level similar to that of the control group after policosanol treatment. Researchers have found that the gut microbiota can regulate the metabolic state and health of the host through pathways such as energy metabolism regulation and release of signaling molecules. According to the previous studies, the balance between *Bacteroides* and *Firmicutes* is considered to be the key factor affecting obesity (35). Furthermore, the altered microbiota can regulate the metabolism of host by regulating the changes of various metabolites. The proportion of *Firmicutes* and *Bacteroidetes* increases, so that more plant derived polysaccharides are decomposed into short fatty acids, which affects the metabolism of carbohydrates and promotes the host’s calories absorption and adipogenesis (6). *Parasutterell* can occupy a specific niche in the intestine and affect the metabolism of aromatic amino acids, bilirubin, succinate and bile acids in the intestine, so as to regulate the metabolism of the host. Therefore, *Parasutterell* is considered to be a core flora but has not been fully studied in recent years (36). In the present study, a combined metabolomics and microbiomics analysis was used to reveal the correlation of metabolites and microbiota. Our results clearly showed that policosanol could significantly influence metabolism in HFD-fed mice. Policosanol significantly increased metabolites associated with the TCA cycle, pyruvate metabolism, glycolysis or gluconeogenesis, and pantothenate and CoA metabolism and significantly inhibited the synthesis of glycerophospholipids. Furthermore, our results clearly revealed that *Bacteroides*, *Allobaculum*, *Staphylococcus*, and *Parasutterella* were participated in succinic acid, pyruvic acid, and critraconic acid regulation. In this study, the results also showed that *Akkmermansia* tended to be increased in HFD + policosanol group, but there was no high correlation in the regulation of metabolites in serum and intestinal contents. Lack of *Akkmermansia* is a key factor in inducing obesity and even diabetes (37). However, studies have shown that compared with living *Akkmermansia*, dead *Akkmermansia* can better regulate host fat content, insulin resistance and hyperlipidemia by releasing Amuc-1100 protein from cell membrane (38, 39).

Lipolysis and thermogenesis process in adipose tissue are important factors regulating fat deposition in mammals. In this study, *Bacteroidetes*, *Firmicutes* in phylum and *Parasutterell* and *Canaidatus_Saccharimonas* in genus level showed high correlation with iWAT and BAT weight and metabolites contents correlated with TCA cycle. Fat in scWAT is more easily metabolized than visceral WAT, which including eWAT and the adipose tissue around liver and heart (40). Compared with visceral WAT, scWAT is more likely to be mobilized, browned, hydrolyzed, oxidized and thermogenerated (41). Therefore, we examined the gene expression associated with lipogenesis and lipolysis in adipose tissue. In obese mice, policosanol did not significantly change the expression of FAS and HSL genes but remarkably increased ATGL gene expression in iWAT. In addition to lipolysis related genes, the genes involved in thermogenesis and adipose “browning” process such as PPARs, UCP-1 and PRDM also play important roles in anti-obesity (14, 27). PPARs, including PPAR α and PPAR γ, regulate the metabolism of liver and adipose tissue, respectively. PPAR α in the liver has the effect of decreasing the level of SREBP-1c, promoting fat thermogenesis and alleviating nonalcoholic fatty liver (42, 43). In addition, we found that PPAR γ regulates fat metabolism in adipose tissue, which was also elevated in scWAT. PPARs are key proteins in thermogenesis. PRDM16 and PGC-1α can combine with PPARs to drive the differentiation of brown adipocytes and promote the expression of UCP-1 (12, 43). In this study, in the HFD+policosanol group, we found that PGC-1α and UCP-1 were increased in iWAT and BAT while PRDM16 was only increased in BAT. These results suggest that in obese mice, policosanol mainly plays a role by increasing the decomposition, oxidation and thermogenesis of lipids.

Based on the findings of this research and predecessors, there are still some limitations to be further studied in the regulation of host metabolism by policosanol through intestinal microbiota. How policosanol regulates intestinal microbiota and how it is metabolized and utilized by intestinal microbiota still remains unclear. In addition, whether the changes of small molecule metabolites in host intestine and serum after treatment with policosanol, such as the increase of polyunsaturated fatty acids and the decrease of TMAO contents, are caused by the changes of intestinal microbiota, which may need to be verified by sterile mouse model. In the future, in-depth research on these aspects may provide a new perspective for the promotion of whole grain food or policosanol as a functional food additive.

## Conclusion

In summary, our findings elucidated that policosanol reduces fat accumulation in obese mice. Furthermore, we found that in obese mice, the metabolites involved in TCA cycle and thermogenesis were highly correlated with *Firmicutes, Bacteroidetes*, and *Candidatus:Sarccharinonasm*, *and Parasutterella*. These findings suggest that policosanol has the potential to be applied as an antihyperlipidemia supplement to improve well-being and health in humans.

## Data Availability Statement

The datasets presented in this study can be found in online repositories. The names of the repositories and accession(s) number can be found below: National Center for Biotechnology Information (NCBI) BioProject, https://www.ncbi.nlm.nih.gov/bioproject/, PRJNA729899 and Figshare, DOI: https://doi.org/10.6084/m9.figshare.14456595.v1.

## Ethics Statement

The animal study was reviewed and approved by Committee of the Institute of Subtropical Agriculture at the Chinese Academy of Science (No. ISA-2020-18).

## Author Contributions

All authors listed have made a substantial, direct, and intellectual contribution to the work and approved it for publication.

## Funding

The project was funded by General Projects of Key Research and Development Plan in Jiangxi Province (20203BBFL63054), Project funded by China Postdoctoral Science Foundation (2020M682108), Guangdong Province enterprise special person special plan project (GDKTP2020054600), Pilot demonstration project for overall rationing system of Jiangxi Academy of Sciences (2021YSBG22008, 2021YSBG50009) and Research and Development Project of Jiangxi Academy of Sciences - Doctoral Fund Project (2020-YYB-01).

## Conflict of Interest

Author HL is employed by Era Biotechnology (Shenzhen) Co., Ltd, China.

The remaining authors declare that the research was conducted in the absence of any commercial or financial relationships that could be construed as a potential conflict of interest.

## Publisher’s Note

All claims expressed in this article are solely those of the authors and do not necessarily represent those of their affiliated organizations, or those of the publisher, the editors and the reviewers. Any product that may be evaluated in this article, or claim that may be made by its manufacturer, is not guaranteed or endorsed by the publisher.
