# Supplementary material for: Integrated Metagenomics and Metabolomics to Reveal the Effects of Policosanol on Modulating the Gut Microbiota and Lipid Metabolism in Hyperlipidemic C57BL/6 Mice
Source: Front Endocrinol (Lausanne). 2021 Oct 11;12:722055. doi: 10.3389/fendo.2021.722055 (PMC8542985; doi:10.3389/fendo.2021.722055)
Supplement: Supplementary file 2 [file Table_1.doc]

**Supplementary information**

**Materials and Methods**

**Table 1 The D12450J diet formula**

| Class description | Ingredients |
| --- | --- |
| protien | Casein, Lactic, 30 Mesh |
| protien | Cystine, L |
| Carbohydrate | Starch, Corn |
| Carbohydrate | Lodex 10 |
| Carbohydrate | Sucrose, Fine Granulated |
| Fiber | Solka Floc, FCC200 |
| Fat | Soybean Oil, USP |
| Fat | Lard |
| Mineral | [S10026B](https://researchdiets.com/formulas/S10026B) |
| Vitamin | Choline Bitartrate |
| Vitamin | [V10001C](https://researchdiets.com/formulas/V10001C) |
| Dye | Dye, Yellow FD&C #5, Alum. Lake 35-42% |
| Dye | Dye, Blue FD&C #1, Alum. Lake 35-42% |
|  |  |
| Caloric Information Physiological Fuel Values | |
| Protein: | 20% Kcal |
| Fat | 10% Kcal |
| Carbohydrate | 70% Kcal |

**Table 2 The high fat diet formula D12492**

| **Ingredients** | Composition ratio | |
| --- | --- | --- |
| D12450J chow diet | 0.38  0.28  0.056  0.108  0.115  0.019  0.02  0.018  0.004  1 | |
| Lard |
| sucrose |
| Whole milk powder |
| casein |
| Microcrystalline cellulose |
| Premix |
| CaHPO3 |
| Stone powder |
| Total |
| **Energy-supply substances** | **Quality ratio (%)** | **Energy supply ratio (%)** |
| Crude protein | 23.25 | 18.14% |
| Crude fat | 34.55 | 60.65 |
| Carbohydrate | 27.2 | 21.22 |
| Total energy | 5127.6 Kcal/kg | 100% |

Table 3 The elution gradient

| Time （min） | A | B |
| --- | --- | --- |
| 0-0.5 | 5% | 95% |
| 0.5-7 | 5-35% | 95-65% |
| 7-8 | 35-60% | 65-40% |
| 8-9 | 60% | 40% |
| 9-9.1 | 60-5% | 40%-95% |
| 9.1-12 | 5% | 95% |

Table 4. Primer information

| Items | Reference sequence ID | Sense | Anti-sense | Product length | Tm (℃） |
| --- | --- | --- | --- | --- | --- |
| FAS | [NM_007987.2](https://www.ncbi.nlm.nih.gov/entrez/viewer.fcgi?db=nucleotide&id=226443048) | TAGAACCTCCAGTCGTGAAACCA | ATCTCATCTATCTTGCCCTCCTTG | 150 | 60 |
| PPARa | [XM_011245516.4](https://www.ncbi.nlm.nih.gov/entrez/viewer.fcgi?db=nucleotide&id=1907110869) | GCGTACGGCAATGGCTTTAT | GAACGGCTTCCTCAGGTTCTT | 57 | 60 |
| PPARγ | [XM_006505737.5](https://www.ncbi.nlm.nih.gov/entrez/viewer.fcgi?db=nucleotide&id=1907171555) | CTGCATCTCCACCTTATTAT | CACAGACTCGGCACTCA | 99 | 60 |
| ATGL | NM_001163689.1 | CAACGCCACTCACATCTACGG | GAAACACGAGTCAGGGAGATGC | 252 | 60 |
| CPT-1a | NM_013495.2 | CGACTCTTCAATACTTCCCGC | TACACGACAATGTGCCTGCTG | 83 | 60 |
| PGC-1a | [NM_008904.2](https://www.ncbi.nlm.nih.gov/entrez/viewer.fcgi?db=nucleotide&id=238018130) | CGAGAAGCGGGAGTCTGAAAG | GAGCAGCGAAAGCGTCACA | 233 | 60 |
| HSL | NM_001039507.2 | GAAGGCACTAGGCGTGATGG | AGACTCTGCTGTGGGCGATG | 148 | 60 |
| UCP1 | NM_009463.3 | AAACAGAAGGATTGCCGAAACT | CTCTGTAGGCTGCCCAATGAA | 206 | 60 |
| PRDM16 | [XM_006539171.5](https://www.ncbi.nlm.nih.gov/entrez/viewer.fcgi?db=nucleotide&id=1907157262) | CAGCACGGTGAAGCCATTC | GCGTGCATCCGCTTGTG | 87 | 60 |
| TNF-α^[1]^ | NM_013693.3 | CGAGTGACAAGCCTGTAGCC | CATGCCGTTGGCCAGGA | 101 | 60 |
| IL-6 ^[1]^ | [NM_001314054.1](https://www.ncbi.nlm.nih.gov/entrez/viewer.fcgi?db=nucleotide&id=930945755) | TCCATCCAGTTGCCTTCTTG | TTCCACGATTTCCCAGAGAAC | 167 | 60 |

Referecne

[1] Guo J, Han X, Tan H , et al. Blueberry Extract Improves Obesity through Regulation of the Gut Microbiota and Bile Acids via Pathways Involving FXR and TGR5[J]. iScience, 2019, 19:676-690.


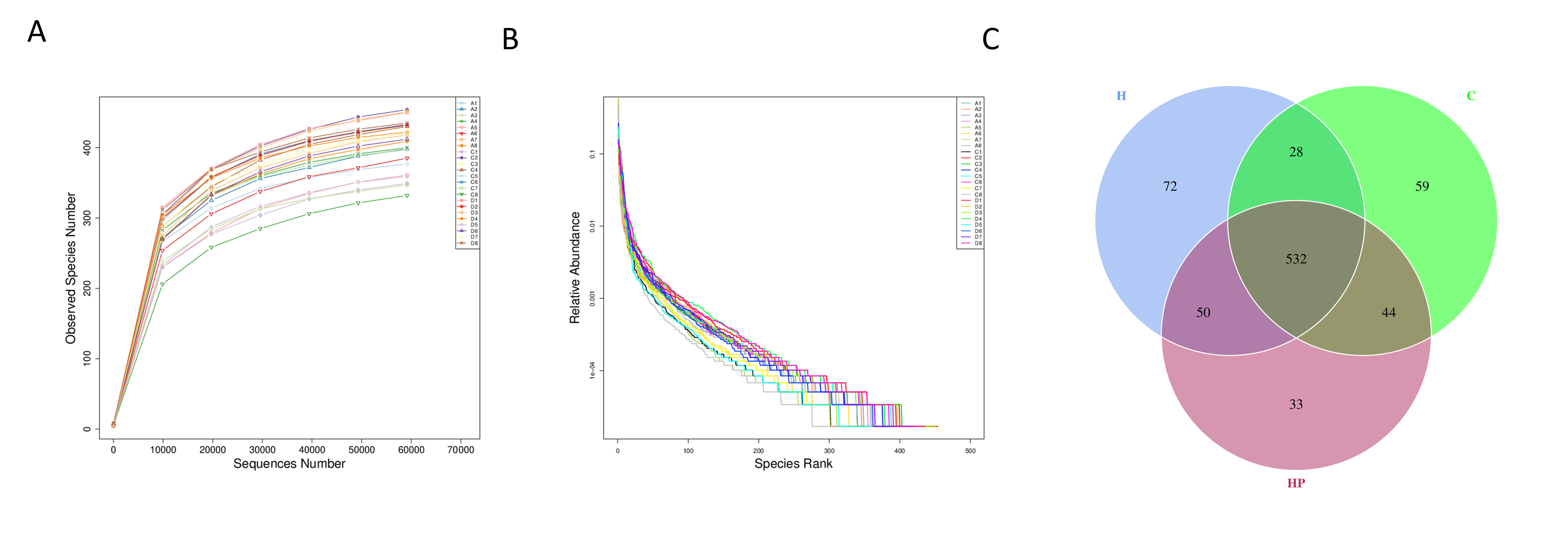


Figure S1. The observed OUTs (A), rarefaction curves (B) and Venn diagram(C).


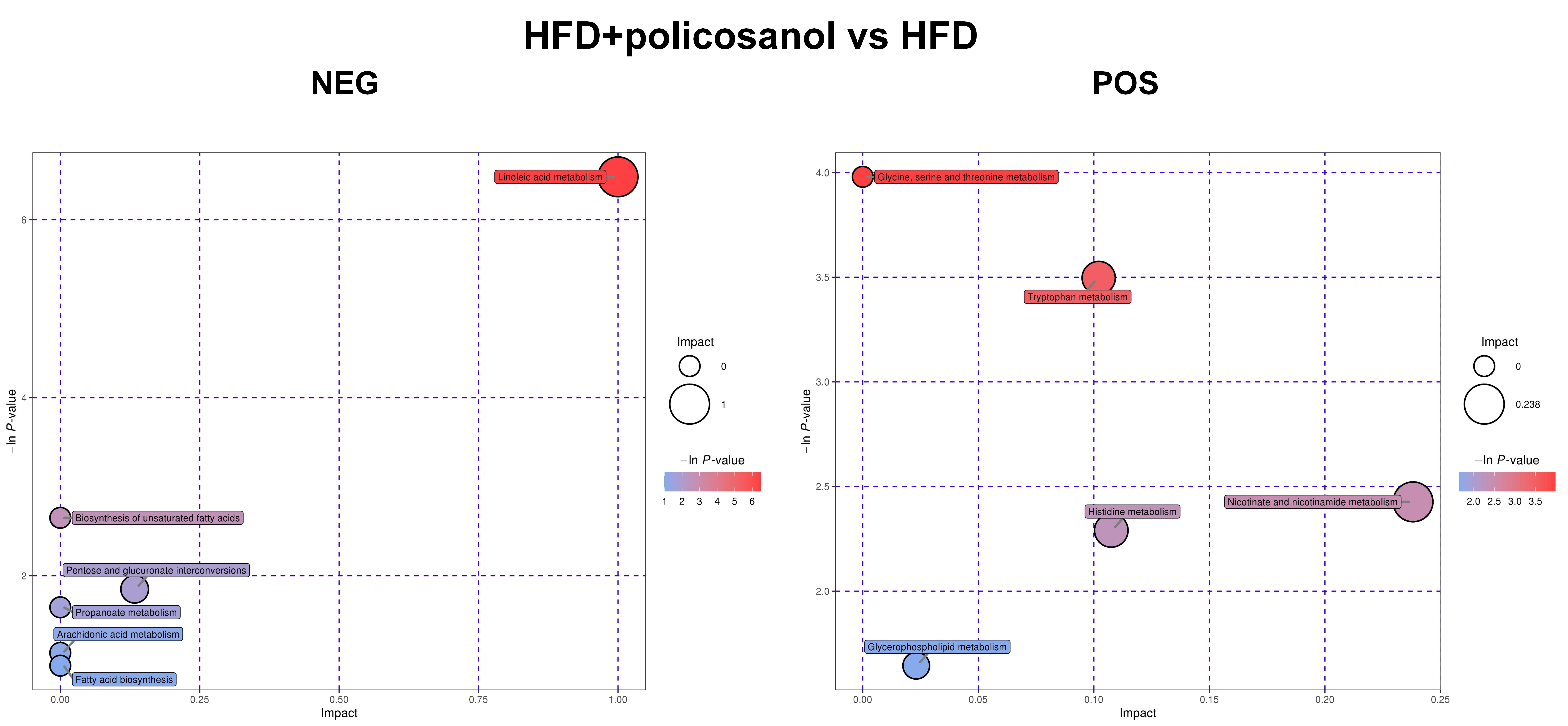


**Figure S2**. The effects of policosanol on the metabolic pathways. (A) Enrichment analysis of KEGG pathways in NEG mode. (B) Enrichment analysis of KEGG pathways in POS mode. NEG: negative scanning mode, POS: positive scanning mode.


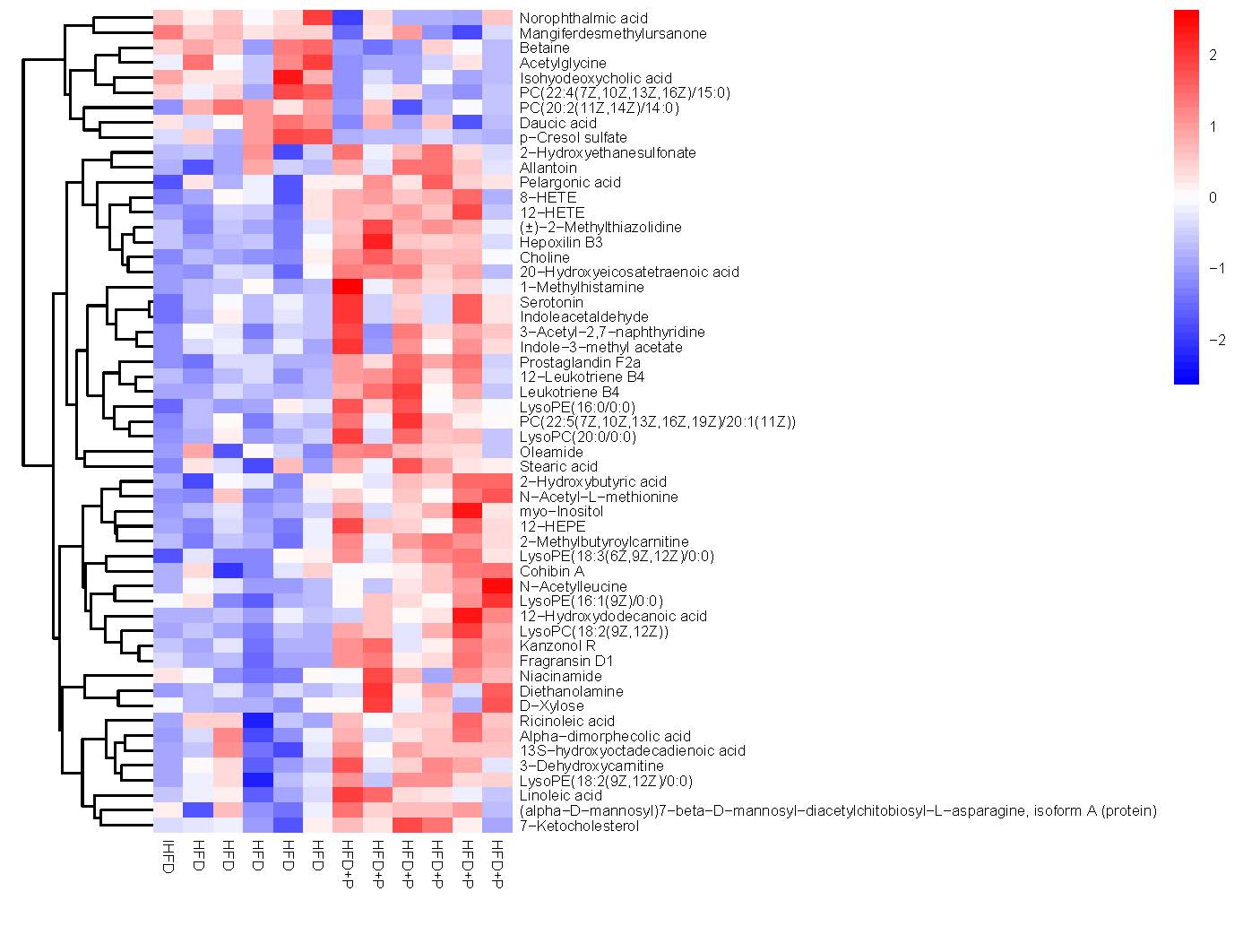


**Figure S3.** Hierarchical clustering analysis of top the 50 metabolites in the cecal contents of mice. The red and blue indicate upregulation and downregulation, respectively.


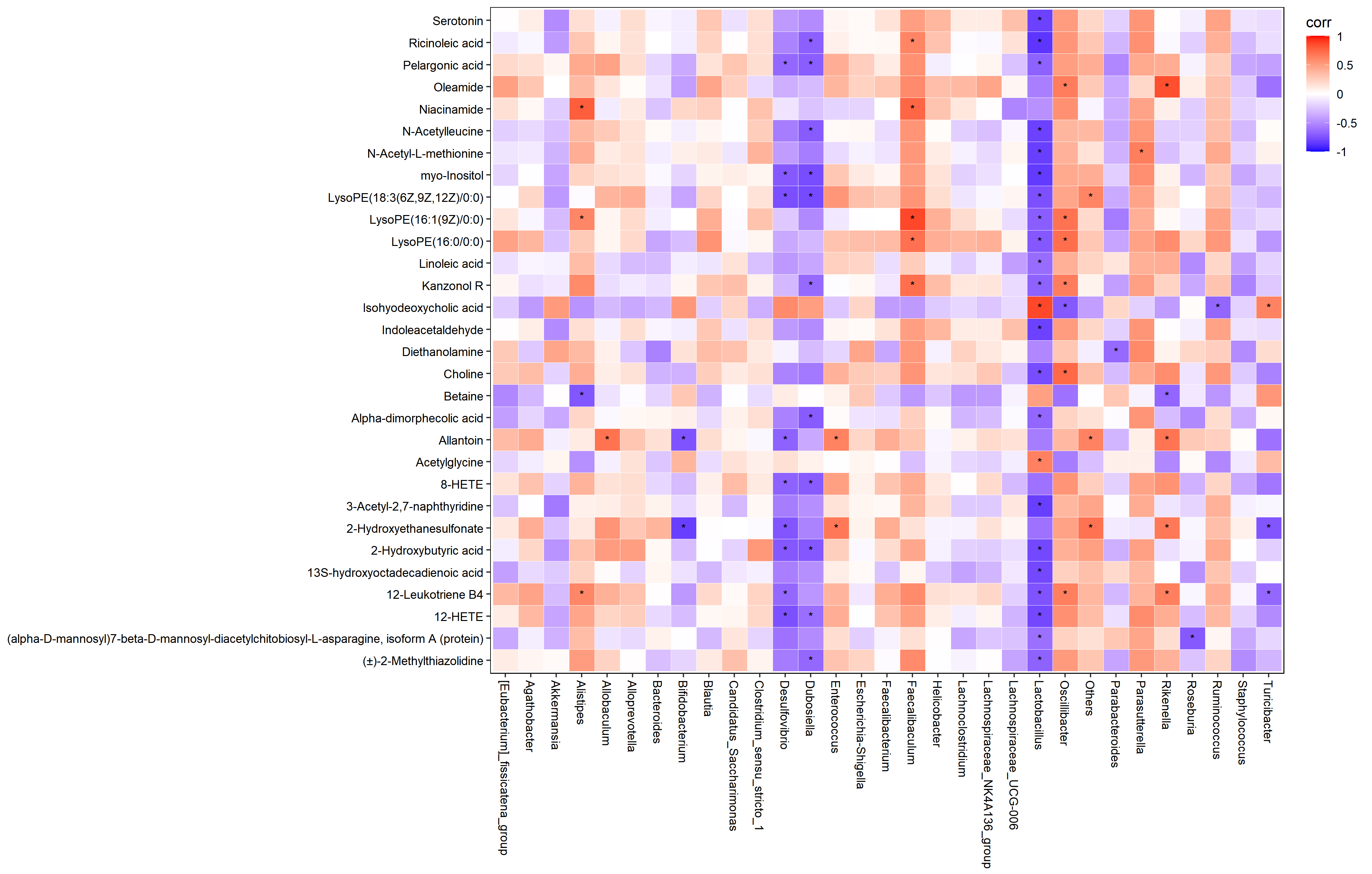


**Figure S4.** Correlation analysis of gut microbiota and cecal content metabolites in HFD and HFD+policosanol mice. Red and blue indicate positive and negative correlations, respectively. *p<0.05.


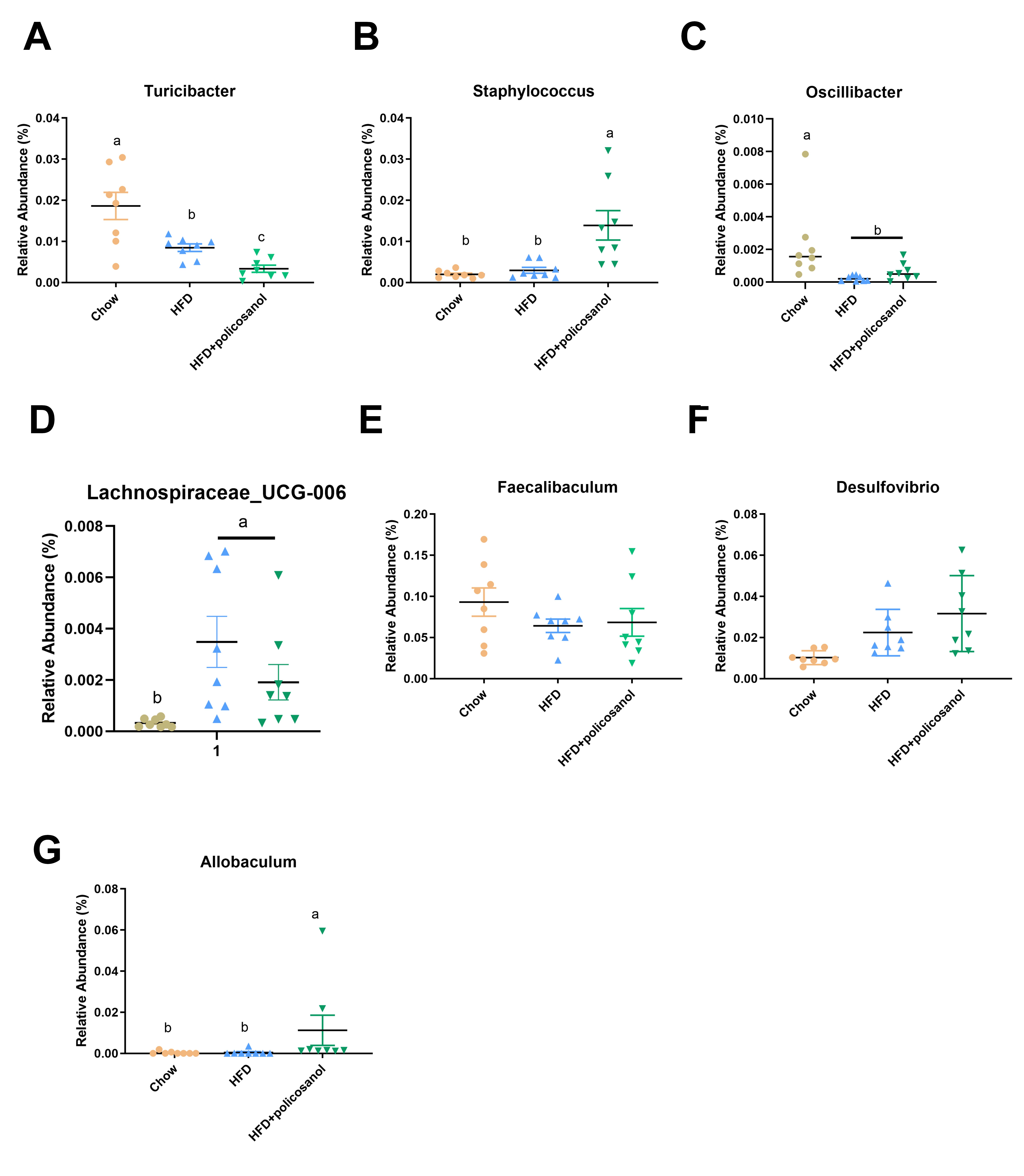


**Figure S5.** The partial fearture taxa identified in Lefse analysis.

Table S5 Differentially expressed serum metabolites between HFD and HFD+policosanol group.

| id | MS2 name | rt | mz | SuperClass | MEAN H+policosanol | MEAN HFD | VIP | P-VALUE | Q-VALUE | FOLD CHANGE | LOG_FOLDCHANGE |
| --- | --- | --- | --- | --- | --- | --- | --- | --- | --- | --- | --- |
| 1 | Caprylic acid | 56.92505 | 143.10706 | Lipids and lipid-like molecules | 4.7842533 | 3.800579 | 1.6796025 | 0.0118106 | 0.1120028 | 1.2588222 | 0.3320746 |
| 2 | Choline | 285.098 | 104.10709 | Organic nitrogen compounds | 53.685007 | 78.503051 | 2.2962503 | 1.894E-05 | 0.0051101 | 0.6838589 | -0.5482295 |
| 3 | Deoxycytidine | 226.088 | 228.0969 | Nucleosides, nucleotides, and analogues | 0.1321992 | 0.0974524 | 1.7825567 | 0.0068422 | 0.0840141 | 1.3565518 | 0.4399442 |
| 4 | Dodecanoic acid | 50.366 | 199.16975 | Lipids and lipid-like molecules | 2.7023854 | 3.1975748 | 1.5289071 | 0.0367133 | 0.1851829 | 0.8451359 | -0.2427447 |
| 5 | Pelargonic acid | 53.4296 | 157.1226 | Lipids and lipid-like molecules | 36.668481 | 29.804584 | 1.6209721 | 0.0228202 | 0.1497489 | 1.2302967 | 0.2990062 |
| 6 | Cytosine | 226.0685 | 112.0505 | Organoheterocyclic compounds | 1.0865017 | 0.8336105 | 1.7535971 | 0.0096819 | 0.1017401 | 1.3033685 | 0.3822451 |
| 7 | Daidzein | 26.80985 | 255.06444 | Phenylpropanoids and polyketides | 0.0072583 | 0.0183382 | 1.899961 | 0.0295292 | 0.1676773 | 0.3958014 | -1.3371514 |
| 8 | Beta-Carboline | 49.509 | 169.07552 | Organoheterocyclic compounds | 0.0870444 | 0.1545301 | 1.7818099 | 0.0247767 | 0.1555043 | 0.5632844 | -0.8280646 |
| 9 | Alpha-dimorphecolic acid | 54.22245 | 295.22791 | Lipids and lipid-like molecules | 0.3827036 | 0.5113835 | 1.8926408 | 0.0022198 | 0.0444604 | 0.7483691 | -0.418178 |
| 10 | Saccharin | 33.4529 | 181.99099 | Organoheterocyclic compounds | 0.1156164 | 0.1894497 | 1.8852027 | 0.0045514 | 0.0646765 | 0.6102751 | -0.7124684 |
| 11 | L-Carnitine | 371.308 | 162.11195 | Organic nitrogen compounds | 29.195301 | 33.309294 | 1.6150955 | 0.0205666 | 0.1427378 | 0.8764911 | -0.1901886 |
| 12 | Trigonelline | 305.456 | 138.05459 | Alkaloids and derivatives | 0.477857 | 0.7845647 | 1.627921 | 0.02361 | 0.1521135 | 0.6090728 | -0.7153135 |
| 13 | 3-Methyl-2-oxovaleric acid | 50.5312 | 129.05497 | Organic acids and derivatives | 18.473415 | 12.797559 | 1.7140602 | 0.0138563 | 0.1201405 | 1.4435108 | 0.5295819 |
| 14 | L-Gulonolactone | 82.43515 | 177.03989 | Organoheterocyclic compounds | 3.0241829 | 2.1189403 | 1.5265655 | 0.0328034 | 0.1753277 | 1.4272148 | 0.5132025 |
| 15 | L-Palmitoylcarnitine | 200.565 | 400.34044 | Lipids and lipid-like molecules | 0.5827578 | 0.6929643 | 1.4750123 | 0.047891 | 0.2096607 | 0.8409638 | -0.2498845 |
| 16 | Riboflavin | 235.4965 | 377.14402 | Organoheterocyclic compounds | 0.0128965 | 0.0179343 | 1.7316179 | 0.0132785 | 0.117987 | 0.7190977 | -0.4757402 |
| 17 | Prostaglandin A2 | 96.4319 | 333.20666 | Lipids and lipid-like molecules | 0.0080377 | 0.0152385 | 1.2896013 | 0.0441768 | 0.2015856 | 0.5274599 | -0.9228666 |
| 18 | Pyruvic acid | 85.43605 | 87.007794 | Organic acids and derivatives | 6.0723103 | 4.4380461 | 1.604605 | 0.0314799 | 0.1720277 | 1.3682396 | 0.4523208 |
| 19 | Mesalazine | 84.9182 | 154.04948 | Benzenoids | 0.4367162 | 0.34553 | 1.4205104 | 0.0434933 | 0.200189 | 1.2639025 | 0.3378851 |
| 20 | Norizalpinin | 25.81755 | 271.05895 | Phenylpropanoids and polyketides | 0.0009714 | 0.0022641 | 1.6233634 | 0.0229902 | 0.1502469 | 0.4290662 | -1.2207277 |
| 21 | 2,3-Dihydroxybutanedioic acid | 31.6468 | 149.00877 | Organic oxygen compounds | 0.1435452 | 0.2770806 | 1.8294571 | 0.036439 | 0.1845279 | 0.5180629 | -0.9488009 |
| 22 | Phosphorylcholine | 496.863 | 184.07259 | Organic nitrogen compounds | 0.398482 | 0.5594272 | 2.0640871 | 0.0003833 | 0.0152815 | 0.7123036 | -0.4894359 |
| 23 | Uridine | 173.9825 | 243.06188 | Nucleosides, nucleotides, and analogues | 6.5427492 | 4.8256796 | 2.0053666 | 0.0019394 | 0.0413057 | 1.3558192 | 0.4391648 |
| 24 | Pantothenic acid | 293.464 | 220.11715 | Organooxygen compounds | 0.0638323 | 0.0926339 | 1.7339989 | 0.0074336 | 0.0881816 | 0.689081 | -0.5372545 |
| 25 | Citraconic acid | 466.796 | 129.01847 | Lipids and lipid-like molecules | 0.6098469 | 0.4046688 | 1.5773151 | 0.0316602 | 0.1724133 | 1.5070271 | 0.5917054 |
| 26 | N-Ethylglycine | 319.396 | 104.07064 | Organic acids and derivatives | 1.1098925 | 0.6595247 | 1.7837322 | 0.0102058 | 0.1044668 | 1.6828673 | 0.7509214 |
| 27 | 2-Oxovaleric acid | 71.2451 | 115.03926 | Organic acids and derivatives | 34.746093 | 24.506403 | 1.7290916 | 0.0078412 | 0.0908931 | 1.4178373 | 0.503692 |
| 28 | Trimethylamine N-oxide | 350.253 | 76.076087 | Organic nitrogen compounds | 0.3160927 | 0.4526985 | 1.6954747 | 0.0132738 | 0.1179687 | 0.6698241 | 0.5182028 |
| 29 | trans-Aconitic acid | 466.731 | 173.0085 | Organic acids and derivatives | 1.2642046 | 0.7859992 | 1.8520282 | 0.0055886 | 0.0741357 | 1.6084043 | 0.6856301 |
| 30 | 5'-Methylthioadenosine | 92.1118 | 298.09589 | Nucleosides, nucleotides, and analogues | 0.1032516 | 0.0598322 | 1.9950092 | 0.0018957 | 0.0407947 | 1.7256871 | 0.7871709 |
| 31 | (±)-2-Methylthiazolidine | 305.456 | 104.05298 | Organoheterocyclic compounds | 0.3290323 | 0.5622386 | 2.2357427 | 3.608E-05 | 0.0061105 | 0.5852183 | -0.7729532 |
| 32 | (9xi,10xi,12xi)-9,10-Dihydroxy-12-octadecenoic acid | 67.1879 | 313.2379 | Lipids and lipid-like molecules | 0.083891 | 0.1237904 | 1.79107 | 0.0077701 | 0.090429 | 0.677686 | -0.5613111 |
| 33 | 3-(4-hydroxyphenyl)-3,4-dihydro-2H-1-benzopyran-7-ol | 27.7452 | 243.10063 | Phenylpropanoids and polyketides | 0.0384465 | 0.0847458 | 2.2543913 | 0.0001587 | 0.0097732 | 0.4536691 | -1.1402876 |
| 34 | Succinic acid | 411.6405 | 117.01852 | Organic acids and derivatives | 7.3038425 | 5.9677017 | 1.7187678 | 0.0157171 | 0.1264184 | 1.2238954 | 0.2914802 |
| 35 | LysoPE(20:5(5Z,8Z,11Z,14Z,17Z)/0:0) | 226.136 | 500.27488 | Lipids and lipid-like molecules | 0.0402825 | 0.0708991 | 1.4171933 | 0.0294125 | 0.1674063 | 0.5681672 | -0.8156127 |
| 36 | N4-Acetylaminobutanal | 66.99735 | 130.08613 | Organic oxygen compounds | 1.2390537 | 0.6102208 | 1.8890048 | 0.0175902 | 0.1327189 | 2.0305006 | 1.0218355 |
| 37 | LysoPE(16:1(9Z)/0:0) | 228.888 | 452.2749 | Lipids and lipid-like molecules | 0.0315018 | 0.0428323 | 1.4836938 | 0.0252454 | 0.1568174 | 0.7354668 | -0.4432679 |
| 38 | N5-(4-Methoxybenzyl)glutamine | 396.74 | 267.13282 | Organic acids and derivatives | 0.0079498 | 0.021982 | 1.9488758 | 0.0281209 | 0.164319 | 0.3616482 | -1.467341 |
| 39 | 2-Pyrocatechuic acid | 27.09605 | 153.01857 | Benzenoids | 0.0789644 | 0.5551132 | 2.0905021 | 0.0041751 | 0.0609265 | 0.1422492 | -2.8135078 |
| 40 | myo-Inositol | 409.5905 | 179.05538 | Organic oxygen compounds | 0.4990362 | 0.5669215 | 1.7346538 | 0.0100179 | 0.1035052 | 0.8802563 | -0.1840044 |
| 41 | Allantoin | 202.9875 | 157.03598 | Organoheterocyclic compounds | 15.975667 | 12.280809 | 2.0766582 | 0.0005957 | 0.0198906 | 1.3008643 | 0.3794705 |
| 42 | Jasmonic acid | 58.3731 | 209.11798 | Lipids and lipid-like molecules | 0.0548754 | 0.0796555 | 1.6374234 | 0.0161636 | 0.1277921 | 0.6889099 | -0.5376129 |
| 43 | Glycerophosphocholine | 405.04 | 258.10913 | Lipids and lipid-like molecules | 15.900692 | 26.226563 | 2.1738317 | 0.0001055 | 0.0083364 | 0.6062819 | -0.7219392 |
| 44 | PI(18:1(9Z)/18:1(9Z)) | 222.4675 | 880.58547 | Lipids and lipid-like molecules | 0.0856223 | 0.1827195 | 2.1577534 | 7.091E-05 | 0.0074286 | 0.4686 | -1.0935712 |
| 45 | PI(20:2(11Z,14Z)/16:0) | 223.347 | 863.55992 | Lipids and lipid-like molecules | 0.0288954 | 0.0667739 | 1.7216853 | 0.0134918 | 0.1187942 | 0.4327342 | -1.208447 |
| 46 | PE(P-18:1(9Z)/20:3(5Z,8Z,11Z)) | 159.123 | 752.55656 | Lipids and lipid-like molecules | 0.5841587 | 0.7199763 | 1.5618149 | 0.0323987 | 0.1742403 | 0.8113583 | -0.301589 |
| 47 | PI(18:1(11Z)/16:1(9Z)) | 224.253 | 835.5317 | Lipids and lipid-like molecules | 0.0184239 | 0.0395383 | 2.1304812 | 0.0002977 | 0.0135377 | 0.4659758 | -1.101673 |
| 48 | Cytidine | 259.3335 | 242.07785 | Nucleosides, nucleotides, and analogues | 0.1916061 | 0.1684917 | 1.9222566 | 0.0031063 | 0.0525622 | 1.137184 | 0.1854657 |
| 49 | PC(24:1(15Z)/14:1(9Z)) | 164.536 | 814.62584 | Lipids and lipid-like molecules | 1.1009397 | 1.5339429 | 1.4541535 | 0.0424859 | 0.1980855 | 0.7177188 | -0.4785093 |
| 50 | Phosphocreatine | 469.359 | 212.04253 | Organic acids and derivatives | 0.0649659 | 0.0483373 | 1.5571664 | 0.0152829 | 0.1250364 | 1.3440121 | 0.4265461 |
| 51 | LysoPE(18:2(9Z,12Z)/0:0) | 226.097 | 478.29185 | Lipids and lipid-like molecules | 1.6067779 | 2.3833964 | 1.8569182 | 0.007727 | 0.0901459 | 0.6741547 | -0.5688484 |
| 52 | Prostaglandin F2a | 103.098 | 353.23336 | Lipids and lipid-like molecules | 0.1985536 | 0.3419117 | 1.598856 | 0.0351778 | 0.1814478 | 0.580716 | -0.7840953 |
| 53 | LysoPC(18:3(6Z,9Z,12Z)) | 221.569 | 518.32085 | Lipids and lipid-like molecules | 1.0463063 | 1.2669768 | 1.6371104 | 0.0198238 | 0.1402465 | 0.8258291 | -0.2760848 |
| 54 | Persicachrome | 214.508 | 385.27193 | Lipids and lipid-like molecules | 0.0795934 | 0.0554935 | 1.3604405 | 0.0439292 | 0.2010824 | 1.4342835 | 0.5203302 |
| 55 | Pseudouridine | 262.1745 | 243.06191 | Nucleosides, nucleotides, and analogues | 0.5982475 | 0.5124885 | 1.5972279 | 0.0230404 | 0.1503932 | 1.1673384 | 0.2232228 |
| 56 | Cohibin A | 226.036 | 549.48553 | Lipids and lipid-like molecules | 0.004212 | 0.0123527 | 1.4604073 | 0.0034002 | 0.05472 | 0.3409739 | -1.5522667 |
| 57 | 3beta,6beta-Dihydroxynortropane | 377.707 | 144.10161 | Alkaloids and derivatives | 0.1624506 | 0.11055 | 1.5445193 | 0.0362445 | 0.1840603 | 1.4694764 | 0.5553022 |
| 58 | 1-Arachidonoylglycerophosphoinositol | 272.593 | 621.3005 | Lipids and lipid-like molecules | 0.1278389 | 0.1597677 | 1.6391813 | 0.0205384 | 0.142645 | 0.8001546 | -0.3216493 |
| 59 | Linamarin | 413.9695 | 248.11199 | Organic oxygen compounds | 0.0249229 | 0.0188876 | 1.5542509 | 0.0185623 | 0.1358437 | 1.3195395 | 0.4000346 |
| 60 | LysoPC(14:0/0:0) | 225.154 | 468.30739 | Lipids and lipid-like molecules | 0.7683673 | 1.2268357 | 2.1374242 | 0.0001576 | 0.0097467 | 0.6263001 | -0.675074 |
| 61 | Oxonantenine | 404.861 | 336.08641 | Alkaloids and derivatives | 0.1585152 | 0.2155677 | 2.00177 | 0.0009783 | 0.0273647 | 0.7353381 | -0.4435203 |
| 62 | PC(16:1(9Z)/P-18:1(11Z)) | 163.718 | 742.57221 |  | 0.0629572 | 0.1032263 | 2.0924583 | 0.0006934 | 0.0217486 | 0.6098943 | -0.7133688 |
| 63 | PC(20:2(11Z,14Z)/15:0) | 39.64145 | 772.57985 | Lipids and lipid-like molecules | 0.6071406 | 0.7525248 | 1.6667683 | 0.0182382 | 0.1348229 | 0.8068048 | -0.3097085 |
| 64 | 2-acetyl-1-alkyl-sn-glycero-3-phosphocholine | 217.1215 | 524.36885 | Lipids and lipid-like molecules | 64.975458 | 84.199706 | 1.61449 | 0.0176469 | 0.1329064 | 0.7716827 | -0.3739203 |
| 65 | PC(20:4(8Z,11Z,14Z,17Z)/20:3(8Z,11Z,14Z)) | 39.529 | 832.58236 | Lipids and lipid-like molecules | 1.4579654 | 1.1916527 | 1.373286 | 0.0419746 | 0.1969971 | 1.2234818 | 0.2909927 |
| 66 | LysoPE(22:6(4Z,7Z,10Z,13Z,16Z,19Z)/0:0) | 222.459 | 526.29166 | Lipids and lipid-like molecules | 2.3993894 | 1.7565365 | 1.6315923 | 0.0218343 | 0.146778 | 1.3659775 | 0.4499338 |
| 67 | SM(d18:1/20:0) | 202.7335 | 759.63314 | Lipids and lipid-like molecules | 0.2214625 | 0.1689602 | 1.4668594 | 0.0495373 | 0.2130698 | 1.3107377 | 0.390379 |
| 68 | LysoPC(20:0/0:0) | 214.4365 | 552.40115 | Lipids and lipid-like molecules | 1.1030084 | 1.5584438 | 1.5838359 | 0.0102617 | 0.1047491 | 0.7077627 | -0.4986623 |
| 69 | Fragransin D1 | 24.9065 | 389.19482 | Lignans, neolignans and related compounds | 0.0475966 | 0.0393823 | 1.4408061 | 0.0480979 | 0.2100956 | 1.208578 | 0.2733106 |
| 70 | Glycerylphosphorylethanolamine | 413.639 | 216.06261 | Organic acids and derivatives | 0.0629109 | 0.0909765 | 1.800611 | 0.004568 | 0.0648384 | 0.6915071 | -0.532184 |
| 71 | N-[(4E,8Z)-1,3-dihydroxyoctadeca-4,8-dien-2-yl]hexadecanamide 1-glucoside | 110.649 | 698.55454 | Lipids and lipid-like molecules | 0.0240946 | 0.0326877 | 1.697009 | 0.0138154 | 0.1199914 | 0.7371141 | -0.4400401 |
| 72 | beta-D-Glucosamine | 413.446 | 214.04803 | Organooxygen compounds | 0.1485013 | 0.2131985 | 1.74115 | 0.0063512 | 0.0803252 | 0.6965402 | -0.5217215 |
| 73 | 5-Aminoimidazole ribonucleotide | 404.934 | 296.06479 | Organic oxygen compounds | 0.2625025 | 0.2974399 | 1.5295306 | 0.0341545 | 0.1788635 | 0.8825395 | -0.1802673 |
| 74 | Cytidine monophosphate | 27.5747 | 324.06069 | Nucleosides, nucleotides, and analogues | 0.0014113 | 0.0035709 | 1.821895 | 0.0100233 | 0.103533 | 0.3952205 | -1.3392703 |

Table S6 Differentially expressed cecal contents metabolites between HFD v.s. HFD+policosanol group.

| MS2 name | MS2 score | rt | mz | SuperClass | type | VIP | P-VALUE | FOLD CHANGE | Mode |
| --- | --- | --- | --- | --- | --- | --- | --- | --- | --- |
| Fragransin D1 | 0.545304308 | 24.9065 | 389.1948191 | Lignans, neolignans and related compounds | forward | 2.585328 | 7.02E-05 | 0.634498 | POS |
| LysoPC(18:2(9Z,12Z)) | 0.659706615 | 91.41405 | 520.3315448 | Lipids and lipid-like molecules | forward | 2.473845 | 0.000341 | 0.619887 | POS |
| Kanzonol R | 0.995090462 | 24.9065 | 371.184342 | Phenylpropanoids and polyketides | forward | 2.466899 | 0.000786 | 0.620537 | POS |
| (±)-2-Methylthiazolidine | 0.954584692 | 305.456 | 104.0529836 | Organoheterocyclic compounds | forward | 2.455217 | 0.000366 | 0.631169 | POS |
| Choline | 0.999995 | 285.098 | 104.1070908 | Organic nitrogen compounds | forward | 2.440557 | 0.000327 | 0.666335 | POS |
| 2-Methylbutyroylcarnitine | 0.846923923 | 263.959 | 246.1692254 | Lipids and lipid-like molecules | forward | 2.316658 | 0.000381 | 0.729583 | POS |
| 12-HEPE | 0.883719462 | 52.49785 | 317.2117686 | Lipids and lipid-like molecules | forward | 2.210492 | 0.00072 | 0.399141 | NEG |
| 12-Leukotriene B4 | 0.979513923 | 79.10345 | 335.223036 | Lipids and lipid-like molecules | forward | 2.20779 | 0.002454 | 0.32281 | NEG |
| Leukotriene B4 | 0.759045 | 80.1397 | 319.2255099 | Lipids and lipid-like molecules | forward | 2.138092 | 0.009622 | 0.098641 | POS |
| 1-Methylhistamine | 0.521407923 | 347.1025 | 126.1023866 | Organic nitrogen compounds | forward | 2.133713 | 0.026225 | 0.535735 | POS |
| Hepoxilin B3 | 0.807112846 | 58.8896 | 335.2229667 | Lipids and lipid-like molecules | forward | 2.090344 | 0.004283 | 0.583605 | NEG |
| myo-Inositol | 0.902950308 | 409.5905 | 179.055381 | Organic oxygen compounds | forward | 2.054819 | 0.010691 | 0.770722 | NEG |
| PC(22:5(7Z,10Z,13Z,16Z,19Z)/20:1(11Z)) | 0.560600846 | 157.464 | 862.62734 | Lipids and lipid-like molecules | forward | 2.048768 | 0.006898 | 0.811553 | POS |
| 12-HETE | 0.941936769 | 52.3522 | 319.2273786 | Lipids and lipid-like molecules | forward | 2.027844 | 0.003948 | 0.597109 | NEG |
| 20-Hydroxyeicosatetraenoic acid | 0.890538231 | 37.9861 | 319.2273295 | Lipids and lipid-like molecules | forward | 2.026794 | 0.002405 | 0.578328 | NEG |
| LysoPC(20:0/0:0) | 0.545458692 | 214.4365 | 552.4011543 | Lipids and lipid-like molecules | forward | 2.022652 | 0.014771 | 0.662367 | POS |
| LysoPE(16:1(9Z)/0:0) | 0.932178 | 228.888 | 452.274903 | Lipids and lipid-like molecules | forward | 1.999799 | 0.007865 | 0.683853 | POS |
| LysoPE(16:0/0:0) | 0.893783231 | 227.04 | 454.2918099 | Lipids and lipid-like molecules | forward | 1.998403 | 0.005966 | 0.841062 | POS |
| Prostaglandin F2a | 0.723302385 | 103.098 | 353.2333559 | Lipids and lipid-like molecules | forward | 1.995231 | 0.001104 | 0.356433 | NEG |
| 3-Dehydroxycarnitine | 0.836298538 | 395.8415 | 146.1171126 | Lipids and lipid-like molecules | forward | 1.992553 | 0.020267 | 0.774034 | POS |
| 8-HETE | 0.962913308 | 52.1356 | 303.2309048 | Lipids and lipid-like molecules | forward | 1.934313 | 0.018704 | 0.688881 | POS |
| Diethanolamine | 0.974335154 | 315.732 | 106.0863512 | Organic nitrogen compounds | forward | 1.930963 | 0.025189 | 0.600821 | POS |
| LysoPE(18:3(6Z,9Z,12Z)/0:0) | 0.903902846 | 227.968 | 476.2743305 | Lipids and lipid-like molecules | forward | 1.913887 | 0.005587 | 0.774915 | POS |
| N-Acetyl-L-methionine | 0.975635846 | 220.2245 | 190.0540201 | Organic acids and derivatives | forward | 1.898302 | 0.007479 | 0.672659 | NEG |
| (alpha-D-mannosyl)7-beta-D-mannosyl-diacetylchitobiosyl-L-asparagine, isoform A (protein) | 0.940919385 | 385.316 | 90.05520255 | Organic acids and derivatives | forward | 1.860951 | 0.032963 | 0.770169 | POS |
| Indoleacetaldehyde | 0.972702538 | 259.749 | 160.075152 | Organoheterocyclic compounds | forward | 1.847773 | 0.029256 | 0.553132 | POS |
| p-Cresol sulfate | 0.55644 | 26.2197 | 187.0063626 | Organic acids and derivatives | forward | 1.835119 | 0.02913 | 7.078037 | NEG |
| 12-Hydroxydodecanoic acid | 0.850369846 | 96.0277 | 215.1650044 | Organic acids and derivatives | forward | 1.831888 | 0.023512 | 0.723264 | NEG |
| Serotonin | 0.993280385 | 259.7765 | 177.1014996 | Organoheterocyclic compounds | forward | 1.818002 | 0.029719 | 0.560276 | POS |
| 13S-hydroxyoctadecadienoic acid | 0.997368538 | 37.9778 | 295.2278101 | Lipids and lipid-like molecules | forward | 1.79165 | 0.023973 | 0.614308 | NEG |
| Mangiferdesmethylursanone | 0.761874077 | 32.20245 | 429.3703633 | Organic oxygen compounds | forward | 1.790945 | 0.046422 | 1.215812 | POS |
| Betaine | 0.999620923 | 291.693 | 118.0861373 | Organic acids and derivatives | forward | 1.785305 | 0.022928 | 1.235075 | POS |
| Isohyodeoxycholic acid | 0.994440308 | 174.4675 | 391.2850469 | Lipids and lipid-like molecules | forward | 1.755948 | 0.010282 | 3.781227 | NEG |
| 2-Hydroxybutyric acid | 0.999994538 | 210.3065 | 103.0392104 | Organic acids and derivatives | forward | 1.743903 | 0.012882 | 0.764445 | NEG |
| Daucic acid | 0.841366077 | 206.339 | 205.0336263 | Organic acids and derivatives | forward | 1.730769 | 0.048675 | 1.746661 | POS |
| LysoPE(18:2(9Z,12Z)/0:0) | 0.726143154 | 226.097 | 478.2918469 | Lipids and lipid-like molecules | forward | 1.721662 | 0.023441 | 0.783211 | POS |
| PC(22:4(7Z,10Z,13Z,16Z)/15:0) | 0.583231462 | 162.847 | 796.5798881 | Lipids and lipid-like molecules | forward | 1.709159 | 0.043259 | 1.596129 | POS |
| N-Acetylleucine | 0.946040462 | 215.499 | 172.0971814 | Organic acids and derivatives | forward | 1.707335 | 0.021609 | 0.677425 | NEG |
| Niacinamide | 0.998731308 | 61.3606 | 123.0552201 | Organoheterocyclic compounds | forward | 1.706939 | 0.048344 | 0.7786 | POS |
| Pelargonic acid | 0.999566923 | 53.4296 | 157.1225953 | Lipids and lipid-like molecules | forward | 1.702369 | 0.013868 | 0.787862 | NEG |
| Oleamide | 0.994008538 | 227.0485 | 282.2781921 | Lipids and lipid-like molecules | forward | 1.69469 | 0.031289 | 0.78236 | POS |
| 3-Acetyl-2,7-naphthyridine | 0.988021154 | 248.6365 | 173.0705158 | Organoheterocyclic compounds | forward | 1.694543 | 0.020496 | 0.648175 | POS |
| Norophthalmic acid | 0.886746692 | 461.4785 | 276.1175936 | Organic acids and derivatives | forward | 1.665962 | 0.032182 | 1.663595 | POS |
| 7-Ketocholesterol | 0.789622 | 34.08495 | 401.3396789 | Lipids and lipid-like molecules | forward | 1.654708 | 0.040898 | 0.639294 | POS |
| Acetylglycine | 0.957996462 | 305.176 | 116.0344732 | Organic acids and derivatives | forward | 1.653429 | 0.021518 | 1.210959 | NEG |
| Linoleic acid | 0.999921154 | 38.94005 | 279.2329512 | Lipids and lipid-like molecules | forward | 1.61264 | 0.034082 | 0.654815 | NEG |
| Indole-3-methyl acetate | 0.512811385 | 254.167 | 190.0857865 | Organoheterocyclic compounds | forward | 1.604032 | 0.024128 | 0.418348 | POS |
| Allantoin | 0.899711923 | 202.9875 | 157.0359764 | Organoheterocyclic compounds | forward | 1.598693 | 0.022437 | 0.84521 | NEG |
| Alpha-dimorphecolic acid | 0.998763615 | 54.22245 | 295.2279146 | Lipids and lipid-like molecules | forward | 1.576121 | 0.048193 | 0.702728 | NEG |
| Ricinoleic acid | 0.933725308 | 54.1549 | 297.2434739 | Lipids and lipid-like molecules | forward | 1.538019 | 0.027529 | 0.73842 | NEG |
| D-Xylose | 0.835481077 | 52.3194 | 149.0449278 | Organic oxygen compounds | forward | 1.521975 | 0.042153 | 0.505448 | NEG |

Table S7 The sources of serum and cecal content metabolites

| MS2 name | MS2 score | rt | mz | SuperClass | KEGG ID | gut | *Mus musculus* |
| --- | --- | --- | --- | --- | --- | --- | --- |
| Choline | 0.999995 | 285.098 | 104.1071 | Organic nitrogen compounds | C00114 | YES | YES |
| Hypoxanthine | 0.999959 | 209.063 | 137.0456 | Organoheterocyclic compounds | C00262 | YES | YES |
| 1-Methylnicotinamide | 0.99991 | 310.597 | 137.0707 | Organoheterocyclic compounds | C02918 |  | YES |
| L-Valine | 0.999818 | 320.315 | 118.0861 | Organic acids and derivatives | C00183 | YES | YES |
| Deoxycytidine | 0.999797 | 226.088 | 228.0969 | Nucleosides, nucleotides, and analogues | C00881 | YES | YES |
| Inosine | 0.999673 | 235.4455 | 269.0872 | Nucleosides, nucleotides, and analogues | C00294 | YES | YES |
| 1-Pyrroline | 0.999647 | 368.5555 | 70.06553 | Organoheterocyclic compounds | C15668 | YES |  |
| Betaine | 0.999621 | 291.693 | 118.0861 | Organic acids and derivatives | C00719 | YES | YES |
| Cytosine | 0.999492 | 226.0685 | 112.0505 | Organoheterocyclic compounds | C00380 | YES |  |
| Sphinganine | 0.999036 | 57.5196 | 302.3041 | Organic nitrogen compounds | C00836 | YES | YES |
| D-Proline | 0.998986 | 525.742 | 116.0706 | Organic acids and derivatives | C00763 | YES |  |
| L-Aspartic acid | 0.998813 | 365.695 | 134.0186 | Organic acids and derivatives | C00049 | YES | YES |
| Niacinamide | 0.998731 | 61.3606 | 123.0552 | Organoheterocyclic compounds | C00153 | YES | YES |
| Benzoic acid | 0.998139 | 322.336 | 123.0439 | Benzenoids | C00180 | YES | YES |
| L-Histidine | 0.998045 | 530.242 | 156.0764 | Organic acids and derivatives | C00135 | YES | YES |
| Creatinine | 0.996681 | 182.6225 | 114.0661 | Organic acids and derivatives | C00791 | YES |  |
| Adenosine | 0.996549 | 183.3735 | 268.1032 | Nucleosides, nucleotides, and analogues | C00212 | YES | YES |
| L-Palmitoylcarnitine | 0.994522 | 200.565 | 400.3404 | Lipids and lipid-like molecules | C02990 |  | YES |
| Trehalose | 0.994358 | 418.093 | 365.1046 | Organic oxygen compounds | C01083 | YES | YES |
| Riboflavin | 0.994004 | 235.4965 | 377.144 | Organoheterocyclic compounds | C00255 | YES | YES |
| L-Phenylalanine | 0.993553 | 279.668 | 166.0857 | Organic acids and derivatives | C00079 | YES | YES |
| L-Methionine | 0.993413 | 304.768 | 150.0579 | Organic acids and derivatives | C00073 | YES | YES |
| Serotonin | 0.99328 | 259.7765 | 177.1015 | Organoheterocyclic compounds | C00780 | YES | YES |
| Urocanic acid | 0.992438 | 312.895 | 139.0499 | Organoheterocyclic compounds | C00785 | YES | YES |
| Creatine | 0.991902 | 368.5165 | 132.0764 | Organic acids and derivatives | C00300 | YES | YES |
| 4-Guanidinobutanoic acid | 0.990401 | 382.324 | 146.092 | Organic acids and derivatives | C01035 | YES | YES |
| 4-O-alpha-D-Galactopyranuronosyl-D-galacturonic acid | 0.98909 | 393.528 | 353.0729 | Organic oxygen compounds | C02273 | YES |  |
| Phosphorylcholine | 0.987879 | 496.863 | 184.0726 | Organic nitrogen compounds | C00588 | YES | YES |
| Pipecolic acid | 0.987061 | 538.3115 | 130.0861 | Organic acids and derivatives | C00408 | YES | YES |
| L-Acetylcarnitine | 0.981793 | 354.9735 | 204.1226 | Lipids and lipid-like molecules | C02571 | YES | YES |
| Pantothenic acid | 0.975327 | 293.464 | 220.1172 | Organooxygen compounds | C00864 | YES | YES |
| L-Glutamic acid | 0.974054 | 422.469 | 148.06 | Organic acids and derivatives | C00025 | YES | YES |
| Thiamine | 0.972977 | 394.7995 | 265.1147 | Organoheterocyclic compounds | C00378 | YES | YES |
| Indoleacetaldehyde | 0.972703 | 259.749 | 160.0752 | Organoheterocyclic compounds | C00637 | YES | YES |
| L-Glutamine | 0.972141 | 393.533 | 147.0761 | Organic acids and derivatives | C00064 | YES | YES |
| Sphingosine | 0.970214 | 78.7637 | 300.2886 | Organic nitrogen compounds | C00319 | YES | YES |
| Histamine | 0.970164 | 383.457 | 112.0868 | Organic nitrogen compounds | C00388 | YES | YES |
| N-Ethylglycine | 0.968032 | 319.396 | 104.0706 | Organic acids and derivatives | C11735 | YES | YES |
| Anserine | 0.966931 | 433.245 | 241.1289 | Organic acids and derivatives | C01262 |  | YES |
| Trimethylamine N-oxide | 0.963739 | 350.253 | 76.07609 | Organic nitrogen compounds | C01104 | YES |  |
| L-Lysine | 0.960934 | 538.4755 | 147.1124 | Organic acids and derivatives | C00047 | YES | YES |
| 5'-Methylthioadenosine | 0.957907 | 92.1118 | 298.0959 | Nucleosides, nucleotides, and analogues | C00170 | YES | YES |
| Citrulline | 0.95585 | 463.2775 | 176.1024 | Organic acids and derivatives | C00327 | YES | YES |
| L-Tyrosine | 0.951191 | 323.045 | 182.0806 | Organic acids and derivatives | C00082 | YES | YES |
| Phytosphingosine | 0.948137 | 61.1679 | 318.2984 | Organic nitrogen compounds | C12144 | YES | YES |
| L-Arginine | 0.945152 | 591.222 | 175.1186 | Organic acids and derivatives | C00062 | YES | YES |
| gamma-Aminobutyric acid | 0.944675 | 391.078 | 104.0707 | Organic acids and derivatives | C00334 | YES | YES |
| Styrene | 0.942346 | 36.8688 | 105.0699 | Benzenoids | C07083 | YES |  |
| Cholesterol | 0.941439 | 26.8605 | 369.3503 | Lipids and lipid-like molecules | C00187 | YES | YES |
| (alpha-D-mannosyl)7-beta-D-mannosyl-diacetylchitobiosyl-L-asparagine, isoform A (protein) | 0.940919 | 385.316 | 90.0552 | Organic acids and derivatives | C01401 | YES |  |
| 3-Indoleacetonitrile | 0.93725 | 52.08365 | 157.0756 | Organoheterocyclic compounds | C02938 | YES |  |
| N4-Acetylaminobutanal | 0.935843 | 66.99735 | 130.0861 | Organic oxygen compounds | C05936 | YES | YES |
| Homocysteine | 0.934411 | 123.268 | 136.0424 | Organic acids and derivatives | C00155 | YES | YES |
| Pyrrolidonecarboxylic acid | 0.93351 | 463.549 | 130.0496 | Organic acids and derivatives | C02237 |  | YES |
| (2R)-2-Hydroxy-2-methylbutanenitrile | 0.929477 | 269.567 | 100.0758 | Organic oxygen compounds | C18796 | YES |  |
| 3-Methylhistidine | 0.925257 | 399.209 | 170.0919 | Organic acids and derivatives | C01152 |  | YES |
| Methylimidazole acetaldehyde | 0.921024 | 207.321 | 125.0707 | Organoheterocyclic compounds | C05827 | YES | YES |
| Ethylbenzene | 0.910573 | 35.7112 | 107.0855 | Benzenoids | C07111 | YES |  |
| Nicotinic acid mononucleotide | 0.902258 | 345.3595 | 256.0805 | Organic oxygen compounds | C01185 | YES | YES |
| 5-Methylcytosine | 0.894803 | 90.1064 | 126.066 | Organoheterocyclic compounds | C02376 | YES |  |
| 5-Aminopentanal | 0.886715 | 312.997 | 102.0915 | Organic oxygen compounds | C12455 | YES | YES |
| L-Threonine | 0.884301 | 373.089 | 120.0655 | Organic acids and derivatives | C00188 | YES | YES |
| Glycerophosphocholine | 0.873131 | 405.04 | 258.1091 | Lipids and lipid-like molecules | C00670 | YES | YES |
| L-4-Hydroxyglutamate semialdehyde | 0.866388 | 493.2585 | 148.06 | Organic acids and derivatives | C05938 | YES | YES |
| PC(22:4(7Z,10Z,13Z,16Z)/15:0) | 0.583231 | 162.847 | 796.5799 | Lipids and lipid-like molecules | C00157 | YES | YES |
| PC(18:3(6Z,9Z,12Z)/18:1(11Z)) | 0.639235 | 86.772 | 782.5665 |  | C00157 | YES | YES |
| PC(16:0/P-16:0) | 0.606777 | 104.706 | 718.5776 | Lipids and lipid-like molecules | C00157 | YES | YES |
| PC(20:2(11Z,14Z)/20:4(5Z,8Z,11Z,14Z)) | 0.646143 | 39.529 | 834.5989 | Lipids and lipid-like molecules | C00157 | YES | YES |
| PC(22:5(4Z,7Z,10Z,13Z,16Z)/20:5(5Z,8Z,11Z,14Z,17Z)) | 0.563537 | 39.4159 | 854.5649 | Lipids and lipid-like molecules | C00157 | YES | YES |
| PC(22:6(4Z,7Z,10Z,13Z,16Z,19Z)/16:1(9Z)) | 0.572166 | 161.96 | 804.5487 | Lipids and lipid-like molecules | C00157 | YES | YES |
| PC(22:5(7Z,10Z,13Z,16Z,19Z)/16:1(9Z)) | 0.642583 | 82.40295 | 806.567 | Lipids and lipid-like molecules | C00157 | YES | YES |
| PC(22:6(4Z,7Z,10Z,13Z,16Z,19Z)/20:2(11Z,14Z)) | 0.575711 | 157.8 | 858.5956 | Lipids and lipid-like molecules | C00157 | YES | YES |
| PC(22:5(4Z,7Z,10Z,13Z,16Z)/P-18:0) | 0.618391 | 156.999 | 820.6187 | Lipids and lipid-like molecules | C00157 | YES | YES |
| PC(18:1(11Z)/14:1(9Z)) | 0.634506 | 61.83835 | 730.5347 | Lipids and lipid-like molecules | C00157 | YES | YES |
| PC(20:2(11Z,14Z)/14:0) | 0.648427 | 60.3923 | 758.5659 | Lipids and lipid-like molecules | C00157 | YES | YES |
| PC(20:4(8Z,11Z,14Z,17Z)/20:3(8Z,11Z,14Z)) | 0.589745 | 39.529 | 832.5824 | Lipids and lipid-like molecules | C00157 | YES | YES |
| PC(18:2(9Z,12Z)/15:0) | 0.569694 | 169.9675 | 744.5497 | Lipids and lipid-like molecules | C00157 | YES | YES |
| PC(24:0/14:1(9Z)) | 0.729081 | 163.766 | 816.6394 | Lipids and lipid-like molecules | C00157 | YES | YES |
| PC(20:1(11Z)/14:1(9Z)) | 0.664661 | 99.02485 | 758.5662 | Lipids and lipid-like molecules | C00157 | YES | YES |
| PC(22:6(4Z,7Z,10Z,13Z,16Z,19Z)/22:6(4Z,7Z,10Z,13Z,16Z,19Z)) | 0.582483 | 154.635 | 878.5666 | Lipids and lipid-like molecules | C00157 | YES | YES |
| PC(18:1(11Z)/15:0) | 0.587753 | 169.966 | 746.5653 | Lipids and lipid-like molecules | C00157 | YES | YES |
| PC(18:2(9Z,12Z)/P-18:1(11Z)) | 0.603271 | 39.48785 | 768.5846 | Lipids and lipid-like molecules | C00157 | YES | YES |
| PC(22:4(7Z,10Z,13Z,16Z)/16:0) | 0.63527 | 62.0733 | 810.597 | Lipids and lipid-like molecules | C00157 | YES | YES |
| PC(24:1(15Z)/14:1(9Z)) | 0.734542 | 164.536 | 814.6258 | Lipids and lipid-like molecules | C00157 | YES | YES |
| PC(22:5(4Z,7Z,10Z,13Z,16Z)/14:0) | 0.748318 | 163.744 | 780.5488 |  | C00157 | YES | YES |
| PC(18:1(9Z)/18:1(11Z)) | 0.800277 | 241.015 | 786.5961 | Lipids and lipid-like molecules | C00157 | YES | YES |
| PC(22:5(7Z,10Z,13Z,16Z,19Z)/18:3(6Z,9Z,12Z)) | 0.556643 | 158.379 | 830.5648 | Lipids and lipid-like molecules | C00157 | YES | YES |
| PC(18:3(6Z,9Z,12Z)/18:0) | 0.64938 | 165.438 | 784.5801 |  | C00157 | YES | YES |
| PC(15:0/15:0) | 0.624368 | 172.645 | 706.5361 | Lipids and lipid-like molecules | C00157 | YES | YES |
| PC(18:2(9Z,12Z)/18:0) | 0.62343 | 130.009 | 786.5956 | Lipids and lipid-like molecules | C00157 | YES | YES |
| PC(18:1(11Z)/14:0) | 0.662259 | 40.2341 | 732.5525 |  | C00157 | YES | YES |
| PC(24:1(15Z)/16:0) | 0.456085 | 143.674 | 844.6812 | Lipids and lipid-like molecules | C00157 | YES | YES |
| PC(20:2(11Z,14Z)/15:0) | 0.602314 | 39.64145 | 772.5798 | Lipids and lipid-like molecules | C00157 | YES | YES |
| PC(20:3(8Z,11Z,14Z)/15:0) | 0.564308 | 40.1981 | 770.5656 | Lipids and lipid-like molecules | C00157 | YES | YES |
| PC(20:5(5Z,8Z,11Z,14Z,17Z)/P-18:1(11Z)) | 0.557725 | 157.413 | 790.5713 | Lipids and lipid-like molecules | C00157 | YES | YES |
| PC(20:5(5Z,8Z,11Z,14Z,17Z)/P-18:0) | 0.614475 | 160.079 | 792.5857 | Lipids and lipid-like molecules | C00157 | YES | YES |
| PC(16:0/15:0) | 0.562232 | 172.653 | 720.5521 | Lipids and lipid-like molecules | C00157 | YES | YES |
| PC(22:2(13Z,16Z)/15:0) | 0.613168 | 39.7366 | 800.6117 | Lipids and lipid-like molecules | C00157 | YES | YES |
| PC(22:6(4Z,7Z,10Z,13Z,16Z,19Z)/20:4(5Z,8Z,11Z,14Z)) | 0.55131 | 156.0455 | 854.565 | Lipids and lipid-like molecules | C00157 | YES | YES |
| PC(20:4(8Z,11Z,14Z,17Z)/P-18:0) | 0.628315 | 160.0565 | 794.6022 | Lipids and lipid-like molecules | C00157 | YES | YES |
| PC(16:1(9Z)/P-18:1(11Z)) | 0.628224 | 163.718 | 742.5722 |  | C00157 | YES | YES |
| PC(20:3(5Z,8Z,11Z)/20:0) | 0.589955 | 161.061 | 840.6376 | Lipids and lipid-like molecules | C00157 | YES | YES |
| PC(22:5(4Z,7Z,10Z,13Z,16Z)/22:1(13Z)) | 0.548122 | 156.378 | 890.6565 | Lipids and lipid-like molecules | C00157 | YES | YES |
| PC(24:1(15Z)/18:3(6Z,9Z,12Z)) | 0.543086 | 157.479 | 866.6585 | Lipids and lipid-like molecules | C00157 | YES | YES |
| PC(18:0/P-16:0) | 0.605495 | 39.65355 | 746.6034 | Lipids and lipid-like molecules | C00157 | YES | YES |
| PC(22:4(7Z,10Z,13Z,16Z)/P-18:0) | 0.5998 | 158.224 | 822.6345 | Lipids and lipid-like molecules | C00157 | YES | YES |
| PC(18:1(11Z)/P-16:0) | 0.595168 | 40.1304 | 744.5872 | Lipids and lipid-like molecules | C00157 | YES | YES |
| PC(22:6(4Z,7Z,10Z,13Z,16Z,19Z)/20:1(11Z)) | 0.572715 | 157.854 | 860.6112 | Lipids and lipid-like molecules | C00157 | YES | YES |
| PC(20:4(5Z,8Z,11Z,14Z)/20:4(8Z,11Z,14Z,17Z)) | 0.546907 | 39.5973 | 830.5642 | Lipids and lipid-like molecules | C00157 | YES | YES |
| PC(22:6(4Z,7Z,10Z,13Z,16Z,19Z)/18:1(11Z)) | 0.613162 | 63.6124 | 832.582 | Lipids and lipid-like molecules | C00157 | YES | YES |
| PC(18:1(9Z)/P-16:0) | 0.557619 | 164.664 | 744.5871 | Lipids and lipid-like molecules | C00157 | YES | YES |
| PC(16:0/16:0) | 0.862517 | 40.3244 | 734.568 | Lipids and lipid-like molecules | C00157 | YES | YES |
| PC(20:3(8Z,11Z,14Z)/14:0) | 0.636005 | 168.18 | 756.5511 | Lipids and lipid-like molecules | C00157 | YES | YES |
| PC(22:5(7Z,10Z,13Z,16Z,19Z)/20:1(11Z)) | 0.560601 | 157.464 | 862.6273 | Lipids and lipid-like molecules | C00157 | YES | YES |
| PC(22:2(13Z,16Z)/16:1(9Z)) | 0.729723 | 60.867 | 812.6136 | Lipids and lipid-like molecules | C00157 | YES | YES |
| N1-Methyl-4-pyridone-3-carboxamide | 0.860593 | 84.70495 | 153.0654 | Organoheterocyclic compounds | C05843 |  | YES |
| 2-(3,4-Dihydroxybenzoyloxy)-4,6-dihydroxybenzoate | 0.843041 | 234.532 | 307.0424 | Phenylpropanoids and polyketides | C04524 | YES |  |
| LysoPC(20:4(5Z,8Z,11Z,14Z)) | 0.623312 | 209.284 | 544.3393 |  | C04230 | YES | YES |
| LysoPC(P-16:0) | 0.772909 | 211.811 | 480.344 | Lipids and lipid-like molecules | C04230 | YES | YES |
| LysoPC(16:1(9Z)/0:0) | 0.522046 | 222.459 | 494.322 | Lipids and lipid-like molecules | C04230 | YES | YES |
| LysoPC(18:1(9Z)) | 0.584561 | 217.969 | 522.3541 | Lipids and lipid-like molecules | C04230 | YES | YES |
| LysoPC(16:0) | 0.642304 | 220.6915 | 496.3392 | Lipids and lipid-like molecules | C04230 | YES | YES |
| LysoPC(17:0) | 0.619244 | 218.895 | 510.3533 | Lipids and lipid-like molecules | C04230 | YES | YES |
| LysoPC(22:0) | 0.61483 | 211.822 | 580.4304 | Lipids and lipid-like molecules | C04230 | YES | YES |
| LysoPC(14:0/0:0) | 0.643129 | 225.154 | 468.3074 | Lipids and lipid-like molecules | C04230 | YES | YES |
| LysoPC(18:3(6Z,9Z,12Z)) | 0.703843 | 221.569 | 518.3208 | Lipids and lipid-like molecules | C04230 | YES | YES |
| LysoPC(22:1(13Z)) | 0.468195 | 212.6945 | 578.4158 |  | C04230 | YES | YES |
| LysoPC(20:0/0:0) | 0.545459 | 214.4365 | 552.4012 | Lipids and lipid-like molecules | C04230 | YES | YES |
| LysoPC(22:6(4Z,7Z,10Z,13Z,16Z,19Z)) | 0.781973 | 215.3245 | 568.3378 | Lipids and lipid-like molecules | C04230 | YES | YES |
| LysoPC(18:2(9Z,12Z)) | 0.659707 | 91.41405 | 520.3315 | Lipids and lipid-like molecules | C04230 | YES | YES |
| LysoPC(24:0) | 0.494784 | 209.918 | 608.4638 | Lipids and lipid-like molecules | C04230 | YES | YES |
| LysoPC(15:0) | 0.57452 | 223.333 | 482.3235 | Lipids and lipid-like molecules | C04230 | YES | YES |
| Indole | 0.766695 | 35.8112 | 118.065 | Organoheterocyclic compounds | C00463 | YES |  |
| D-Alanine | 0.762405 | 367.595 | 90.05519 | Organic acids and derivatives | C00133 | YES |  |
| Imidazole-4-acetaldehyde | 0.759492 | 76.1504 | 111.0553 | Organoheterocyclic compounds | C05130 | YES | YES |
| Leukotriene B4 | 0.759045 | 80.1397 | 319.2255 | Lipids and lipid-like molecules | C02165 |  | YES |
| Aminoacetone | 0.743519 | 84.8955 | 74.0604 | Organic oxygen compounds | C01888 | YES | YES |
| Phosphocreatine | 0.730951 | 469.359 | 212.0425 | Organic acids and derivatives | C02305 | YES | YES |
| 2-Phenylacetamide | 0.730232 | 322.713 | 136.0755 | Benzenoids | C02505 | YES |  |
| Monoethylglycinexylidide | 0.71675 | 49.2093 | 207.1585 | Organic acids and derivatives | C16561 | YES | YES |
| SM(d18:0/18:1(9Z)) | 0.592016 | 184.3045 | 731.6023 | Lipids and lipid-like molecules | C00550 | YES | YES |
| SM(d18:1/18:1(9Z)) | 0.579208 | 204.4515 | 729.587 | Lipids and lipid-like molecules | C00550 | YES | YES |
| SM(d18:1/20:0) | 0.550885 | 202.7335 | 759.6331 | Lipids and lipid-like molecules | C00550 | YES | YES |
| SM(d18:1/22:0) | 0.54949 | 201.584 | 787.6628 | Lipids and lipid-like molecules | C00550 | YES | YES |
| SM(d18:1/24:1(15Z)) | 0.532441 | 181.6865 | 813.6798 | Lipids and lipid-like molecules | C00550 | YES | YES |
| SM(d18:1/16:0) | 0.702476 | 206.4335 | 703.5716 | Lipids and lipid-like molecules | C00550 | YES | YES |
| Formiminoglutamic acid | 0.697678 | 423.465 | 175.0708 | Organic acids and derivatives | C00439 | YES | YES |
| Ornithine | 0.686339 | 527.7805 | 133.0969 | Organic acids and derivatives | C00077 | YES | YES |
| Linamarin | 0.675554 | 413.9695 | 248.112 | Organic oxygen compounds | C01594 | YES |  |
| L-Octanoylcarnitine | 0.671978 | 229.852 | 288.2161 | Lipids and lipid-like molecules | C02838 |  | YES |
| D-Glutamine | 0.664334 | 464.32 | 147.076 | Organic acids and derivatives | C00819 | YES | YES |
| PE(20:5(5Z,8Z,11Z,14Z,17Z)/P-18:0) | 0.661115 | 40.2019 | 750.5381 | Lipids and lipid-like molecules | C00350 | YES | YES |
| PE(16:0/18:2(9Z,12Z)) | 0.510508 | 172.644 | 716.5206 | Lipids and lipid-like molecules | C00350 | YES | YES |
| 5-Aminopentanoic acid | 0.538207 | 404.703 | 118.0861 | Organic acids and derivatives | C00431 | YES |  |
| Glycerylphosphorylethanolamine | 0.535496 | 413.639 | 216.0626 | Organic acids and derivatives | C01233 | YES | YES |
| 1-Methylhistamine | 0.521408 | 347.1025 | 126.1024 | Organic nitrogen compounds | C05127 | YES | YES |
| 5-Aminoimidazole ribonucleotide | 0.50841 | 404.934 | 296.0648 | Organic oxygen compounds | C03373 | YES | YES |
| Cytidine monophosphate | 0.421221 | 27.5747 | 324.0607 | Nucleosides, nucleotides, and analogues | C00055 | YES | YES |
| Malonic acid | 1 | 109.7415 | 103.0027 | Organic acids and derivatives | C00383 | YES | YES |
| Palmitoleic acid | 1 | 685.891 | 253.2171 | Lipids and lipid-like molecules | C08362 |  | YES |
| Sarcosine | 1 | 367.1565 | 88.03948 | Organic acids and derivatives | C00213 | YES | YES |
| Caprylic acid | 1 | 56.92505 | 143.1071 | Lipids and lipid-like molecules | C06423 | YES | YES |
| 2-Hydroxybutyric acid | 0.999995 | 210.3065 | 103.0392 | Organic acids and derivatives | C05984 | YES | YES |
| Oleic acid | 0.999971 | 39.00795 | 281.2484 | Lipids and lipid-like molecules | C00712 | YES | YES |
| Linoleic acid | 0.999921 | 38.94005 | 279.233 | Lipids and lipid-like molecules | C01595 | YES | YES |
| Arachidonic acid | 0.999824 | 42.084 | 303.2327 | Lipids and lipid-like molecules | C00219 | YES | YES |
| Dodecanoic acid | 0.999712 | 50.366 | 199.1698 | Lipids and lipid-like molecules | C02679 | YES | YES |
| L-Proline | 0.999279 | 329.93 | 114.0552 | Organic acids and derivatives | C00148 | YES | YES |
| (R)-3-Hydroxybutyric acid | 0.999067 | 252.26 | 103.0392 | Organic acids and derivatives | C01089 | YES | YES |
| 3-Hydroxybutyric acid | 0.55858 | 290.506 | 103.0392 | Organic acids and derivatives | C01089 | YES | YES |
| Uracil | 0.998232 | 82.05535 | 111.0191 | Organoheterocyclic compounds | C00106 | YES | YES |
| Chenodeoxycholic acid | 0.99818 | 155.103 | 391.2851 | Lipids and lipid-like molecules | C02528 | YES | YES |
| Taurine | 0.997844 | 316.229 | 124.0065 | Organic acids and derivatives | C00245 | YES | YES |
| Phenylacetylglycine | 0.997736 | 207.821 | 192.0662 | Organic acids and derivatives | C05598 |  | YES |
| Stearidonic acid | 0.997612 | 48.6784 | 275.2012 | Lipids and lipid-like molecules | C16300 |  | YES |
| Gamma-Linolenic acid | 0.997462 | 97.6306 | 277.2168 | Lipids and lipid-like molecules | C06426 | YES | YES |
| Docosahexaenoic acid | 0.997176 | 38.9511 | 327.2325 | Lipids and lipid-like molecules | C06429 | YES | YES |
| Thymidine | 0.997032 | 94.24535 | 241.0828 | Nucleosides, nucleotides, and analogues | C00214 | YES | YES |
| Pyroglutamic acid | 0.996087 | 323.496 | 128.0345 | Organic acids and derivatives | C01879 | YES | YES |
| L-Gulonolactone | 0.995968 | 82.43515 | 177.0399 | Organoheterocyclic compounds | C01040 | YES | YES |
| Vanillin | 0.995701 | 51.38885 | 151.0395 | Benzenoids | C00755 | YES |  |
| Glutaric acid | 0.995307 | 412.373 | 131.0341 | Organic acids and derivatives | C00489 | YES |  |
| Taurocholic acid | 0.994335 | 224.7205 | 514.2849 | Lipids and lipid-like molecules | C05122 | YES | YES |
| Benzoic acid | 0.994304 | 125.632 | 121.0286 | Benzenoids | C00180 | YES | YES |
| Succinic acid semialdehyde | 0.993889 | 222.0595 | 101.0235 | Lipids and lipid-like molecules | C00232 | YES | YES |
| But-2-enoic acid | 0.992753 | 176.879 | 85.02855 | Lipids and lipid-like molecules | C01771 | YES |  |
| Pyruvic acid | 0.992546 | 85.43605 | 87.00779 | Organic acids and derivatives | C00022 | YES | YES |
| 2-Hydroxyethanesulfonate | 0.990984 | 170.395 | 124.9907 | Organic acids and derivatives | C05123 | YES |  |
| Citric acid | 0.990514 | 584.521 | 191.019 | Organic acids and derivatives | C00158 | YES | YES |
| 4-Hydroxybenzaldehyde | 0.989703 | 38.8231 | 121.0286 | Organic oxygen compounds | C00633 | YES |  |
| Xanthine | 0.98916 | 233.128 | 151.0256 | Organoheterocyclic compounds | C00385 | YES | YES |
| Terephthalic acid | 0.986328 | 384.916 | 165.0186 | Benzenoids | C06337 | YES |  |
| Perillic acid | 0.984802 | 67.74295 | 165.0914 | Lipids and lipid-like molecules | C11924 | YES | YES |
| Uridine | 0.98328 | 173.9825 | 243.0619 | Nucleosides, nucleotides, and analogues | C00299 | YES | YES |
| D-Xylitol | 0.979582 | 100.1818 | 151.0604 | Organic oxygen compounds | C00379 | YES | YES |
| 12-Leukotriene B4 | 0.979514 | 79.10345 | 335.223 | Lipids and lipid-like molecules | C04853 |  | YES |
| L-Phenylalanine | 0.976298 | 311.304 | 164.071 | Organic acids and derivatives | C00079 | YES | YES |
| Estrone glucuronide | 0.970364 | 36.9943 | 445.1865 | Lipids and lipid-like molecules | C11133 |  | YES |
| Citraconic acid | 0.970029 | 466.796 | 129.0185 | Lipids and lipid-like molecules | C02226 | YES |  |
| L-Tyrosine | 0.968989 | 323.558 | 180.0659 | Organic acids and derivatives | C00082 | YES | YES |
| L-Valine | 0.966588 | 320.7585 | 116.0708 | Organic acids and derivatives | C00183 | YES | YES |
| Maleic acid | 0.965693 | 402.4695 | 115.0029 | Organic acids and derivatives | C01384 | YES |  |
| m-Coumaric acid | 0.965666 | 57.8085 | 163.0393 | Phenylpropanoids and polyketides | C12621 | YES |  |
| Creatinine | 0.965054 | 181.357 | 112.0507 | Organic acids and derivatives | C00791 | YES |  |
| Prostaglandin D2 | 0.964602 | 47.8094 | 351.2208 | Lipids and lipid-like molecules | C00696 | YES | YES |
| Creatine | 0.962132 | 368.087 | 130.0614 | Organic acids and derivatives | C00300 | YES | YES |
| trans-Aconitic acid | 0.962006 | 466.731 | 173.0085 | Organic acids and derivatives | C02341 | YES |  |
| Hippuric acid | 0.961268 | 213.971 | 178.0502 | Benzenoids | C01586 | YES | YES |
| Succinic acid | 0.943438 | 411.6405 | 117.0185 | Organic acids and derivatives | C00042 | YES | YES |
| D-Glutamine | 0.939158 | 394.0175 | 145.0612 | Organic acids and derivatives | C00819 | YES | YES |
| Inosine | 0.930672 | 235.945 | 267.0729 | Nucleosides, nucleotides, and analogues | C00294 | YES | YES |
| 4-Pyridoxic acid | 0.927758 | 45.9741 | 182.0454 | Organoheterocyclic compounds | C00847 | YES | YES |
| L-Glutamine | 0.918092 | 442.51 | 145.0612 | Organic acids and derivatives | C00064 | YES | YES |
| Trehalose | 0.91176 | 385.022 | 341.1093 | Organic oxygen compounds | C01083 | YES | YES |
| 2-Pyrocatechuic acid | 0.90793 | 27.09605 | 153.0186 | Benzenoids | C00196 | YES |  |
| Pantothenic acid | 0.90617 | 293.3595 | 218.1028 | Organooxygen compounds | C00864 | YES | YES |
| myo-Inositol | 0.90295 | 409.5905 | 179.0554 | Organic oxygen compounds | C00137 | YES | YES |
| Deoxyuridine | 0.896784 | 111.394 | 227.067 | Nucleosides, nucleotides, and analogues | C00526 | YES | YES |
| Xanthosine | 0.893767 | 335.485 | 283.0679 | Nucleosides, nucleotides, and analogues | C01762 | YES | YES |
| 20-Hydroxyeicosatetraenoic acid | 0.890538 | 37.9861 | 319.2273 | Lipids and lipid-like molecules | C14748 |  | YES |
| Imidazoleacetic acid | 0.886473 | 95.0219 | 125.0349 | Organoheterocyclic compounds | C02835 | YES | YES |
| 3-Sulfinato-L-alaninate | 0.865051 | 198.235 | 152.0015 | Organic acids and derivatives | C00606 | YES | YES |
| Hydrogen phosphate | 0.861763 | 183.348 | 96.96894 | Homogeneous non-metal compounds | C01293 |  | YES |
| Indoleacetaldehyde | 0.853905 | 35.2646 | 158.0603 | Organoheterocyclic compounds | C00637 | YES | YES |
| alpha-Tocopherol | 0.853888 | 32.616 | 429.3737 | Lipids and lipid-like molecules | C02477 | YES |  |
| Hydroxypyruvic acid | 0.84878 | 413.222 | 103.0028 | Organic acids and derivatives | C00168 | YES | YES |
| Anserine | 0.84051 | 433.6895 | 239.1149 | Organic acids and derivatives | C01262 |  | YES |
| 2-Ketobutyric acid | 0.839577 | 393.646 | 101.0236 | Organic acids and derivatives | C00109 | YES | YES |
| D-Xylose | 0.835481 | 52.3194 | 149.0449 | Organic oxygen compounds | C00181 | YES | YES |
| Hypoxanthine | 0.833293 | 182.501 | 135.0305 | Organoheterocyclic compounds | C00262 | YES | YES |
| Sedoheptulose | 0.831567 | 64.0996 | 209.0662 | Organic oxygen compounds | C02076 | YES | YES |
| Cholesterol sulfate | 0.817349 | 27.1001 | 465.3045 | Lipids and lipid-like molecules | C18043 |  | YES |
| Eicosapentaenoic acid | 0.791338 | 38.8731 | 301.2167 | Lipids and lipid-like molecules | C06428 | YES | YES |
| L-Allothreonine | 0.786221 | 393.4665 | 118.0502 | Organic acids and derivatives | C05519 | YES | YES |
| Deoxyribose 5-phosphate | 0.766941 | 209.187 | 213.0165 | Organic oxygen compounds | C00673 | YES | YES |
| Cytidine | 0.752027 | 259.3335 | 242.0779 | Nucleosides, nucleotides, and analogues | C00475 | YES | YES |
| LysoPA(18:1(9Z)/0:0) | 0.740768 | 221.013 | 435.2523 | Lipids and lipid-like molecules | C00416 | YES | YES |
| LysoPA(16:0/0:0) | 0.715248 | 221.844 | 409.2362 | Lipids and lipid-like molecules | C00416 | YES | YES |
| Prostaglandin F2a | 0.723302 | 103.098 | 353.2334 | Lipids and lipid-like molecules | C00639 | YES | YES |
| L-Homoserine | 0.710223 | 372.6255 | 118.0502 | Organic acids and derivatives | C00263 | YES |  |
| Stearic acid | 0.704537 | 39.08475 | 283.2634 | Lipids and lipid-like molecules | C01530 | YES | YES |
| Pseudouridine | 0.689204 | 262.1745 | 243.0619 | Nucleosides, nucleotides, and analogues | C02067 | YES | YES |
| Ketoleucine | 0.677329 | 172.8785 | 129.055 | Organic acids and derivatives | C00233 | YES | YES |
| Cortisone | 0.545341 | 148.6305 | 359.1892 | Lipids and lipid-like molecules | C00762 | YES | YES |
| AICAR | 0.508513 | 325.3525 | 337.0543 | Nucleosides, nucleotides, and analogues | C04677 | YES | YES |
| Dihydrolipoate | 0.442554 | 71.4042 | 207.0504 | Lipids and lipid-like molecules | C02147 | YES |  |
